# Novel metal sites revealed by spectroscopic and structural characterization of the ferric uptake regulator from *Acidithiobacillus ferrooxidans*

**DOI:** 10.1016/j.csbj.2025.02.017

**Published:** 2025-02-19

**Authors:** Yerko Argandoña, Andrea Olivos, Patricia Obando, Francisco Imas, Ehmke Pohl, Raquel Quatrini, Mauricio Arenas-Salinas

**Affiliations:** a Centro de Bioinformática, Simulación y Modelado (CBSM). Facultad de Ingeniería. Universidad de Talca, Talca 346 5548, Chile; b Centro Científico y Tecnológico de Excelencia Ciencia & Vida, Santiago, Chile; c Facultad de Medicina y Ciencia, Universidad San Sebastián, Providencia, Santiago 7510157, Chile; d Department of Chemistry & School of Biological and Biomedical Sciences, Biophysical Sciences Institute, Durham University, Durham DH1 3LE, UK

**Keywords:** Fur, Ferric uptake regulator, Iron-sulfur cluster, Site-directed mutagenesis, Extremophile, *Acidithiobacillia*, Iron homeostasis, Biomining, Biotechnology

## Abstract

*Acidithiobacillus ferrooxidans* (*Af.*) is a microorganism of significant biotechnological interest that thrives in acidic environments with very high concentrations of soluble iron. Understanding the molecular mechanisms that enable its survival in these extreme conditions is of great scientific relevance and practical importance for bioleaching of precious metals. Central to its metabolism is the Ferric Uptake Regulator (Fur), a protein that regulates iron homeostasis and responses to oxidative stress in bacteria. Using a combination of bioinformatics, experimental, and spectroscopic methodologies, this study identified and characterized the metal binding sites and cofactors relevant to AfFur´s function. Three metal-binding sites became evident, two of which are very similar to those found in other members of the superfamily. The third site, formed by four cysteine residues in a configuration CX_2_C-X_n_-CX_8_C, stably binds an iron-sulfur cluster. Site-directed mutagenesis coupled with Electrophoretic Mobility Shift Assays underscored the essentiality of these cysteines for AfFur’s functionality, particularly in DNA binding. Altogether, the findings from this research suggest the presence of an iron-sulfur cluster, which may play a role in fine-tuning iron sensing, particularly adapted to the unique environment of *Acidithiobacillus ferrooxidans*.

## Graphical Abstract

**Figure:**
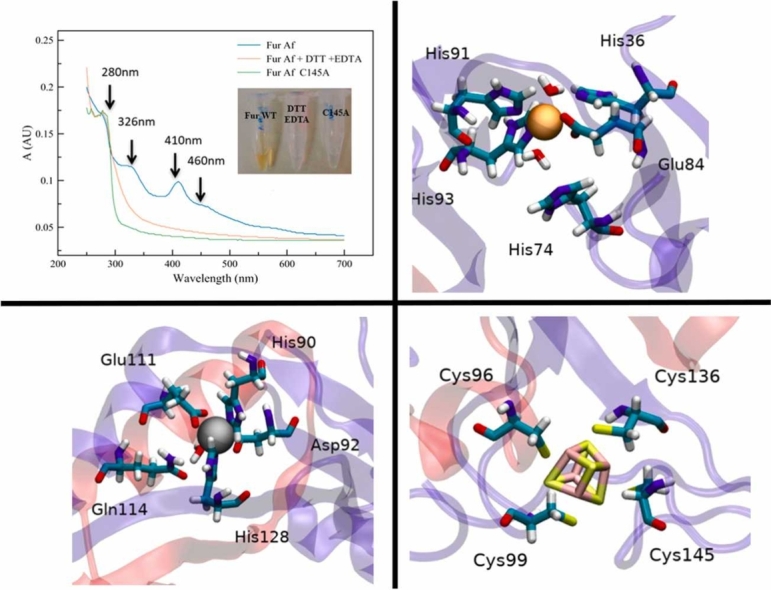

## 1 Introduction

The Ferric Uptake Regulator (Fur) is the main regulator involved in the transcriptional control of iron homeostasis related genes in bacteria. It was first described in *Salmonella typhimurium* [1] and thereafter identified in the vast majority of bacteria. The Fur protein from *Escherichia coli* (EcFur) is one of the best studied representatives of this protein family. EcFur has been acknowledged to tightly regulate the expression of more than 100 genes implicated in iron transport and storage, reactive oxygen species (ROS) resistance, and pathogenicity, among other processes, in response to iron availability [2], [3], [4]. In other non-model microorganisms, Fur regulates a similar number of genes [5], [6], [7], [8]. Based on the number of genes under its control, Fur is defined as a global regulator implicated in adjusting cellular physiology in response to iron concentrations.

Analysis of EcFur initially revealed the presence of two distinct metal-binding sites: a regulatory site for iron and a structural site for zinc [9], [10]. The regulatory iron-binding site, Site 1, is coordinated by His-87, Asp-89, Glu-108, and His-125. In contrast, the structural zinc-binding site, Site 2, involves coordination by His-33, Glu-81, His-88, and His-90 [11]. Recent discoveries have further identified a third metal-binding site in EcFur, which accommodates a [2Fe-2S] cluster. This cluster is coordinated by Cys-93, Cys-96, and Cys-113[12]. The regulatory metal ions determine Fur's ability to bind to specific DNA sequences [9]. The second metal-binding site demonstrates a high affinity for Zn^2 +^ and is essential for the structural stabilization of the protein [10]. In the third site, the [2Fe-2S] cluster binds to regulate intracellular iron homeostasis by responding to elevated levels of free intracellular iron in *E. coli* cells [11].

The apo-protein EcFur and its form containing only the structural Zn^2+^ lose the ability to bind DNA. Activation of EcFur occurs only upon incorporation of a second metal ion at the regulatory site. Research focused on characterizing the metal-binding sites of EcFur and some of its orthologs has demonstrated that Fur can be activated by various divalent metals, including Co(II), Fe(II)/Fe(III), Zn(II), and Mn(II) [13], [14], [15], [16], [17].

As a global regulator of gene expression, Fur is different from other canonical bacterial regulatory systems in both mechanistic and structural aspects, acting as both a positive and a negative regulator [18], [19]. Another notable feature of Fur is that it regulates the expression of virulence genes in multiple pathogenic bacteria such as *Salmonella enterica* [20], *Pseudomonas aeruginosa* [21], *Mycobacterium tuberculosis* [22], *Vibrio cholera* [23], and *Helicobacter pylori* [24], making it an attractive putative therapeutic target for antibacterial drugs [25], [26], [27], [28]. However, to achieve such a goal, it is indispensable to characterize Fur protein family representatives with distinct properties and determine their molecular mechanisms.

*Acidithiobacillus ferrooxidans* is an acidophilic, preferentially aerobic bacterium pertaining to the *Acidithiobacillia* class [29] and one of few known microorganisms capable of using ferrous iron (Fe^2+^) as an electron and energy source to fix CO_2_ and N_2_ under extreme acid conditions [30]. It is one of the most studied acidophiles due to its relevance in bioleaching and potential importance in astrobiology, and because of the extreme conditions of its habitat. Recently, it has grown in importance due to its ability to produce magnetosomes, molecular structures of great interest for drug development and nanotechnology [31], [32].

Since Fe^2+^ provides a limited amount of energy to cover bacterial growth requirements, *A. ferrooxidans* (Af.) is confronted with the task of oxidizing large quantities of Fe^2+^ to Fe^3+^, leading to high O_2_ consumption and the progressive accumulation of Fe^3+^ ions in its environment [33]. However, iron excess under aerobic conditions is extremely toxic to any organism by producing highly reactive oxygen species [34]. Therefore, the possibility that *A. ferrooxidans* has developed new mechanisms for maintaining iron homeostasis and controlling oxidative stress has motivated several studies [35], [36]. These, and other studies have established the occurrence and general conservation of the iron responsive Fur regulator in *A. ferrooxidans* (AfFur) and members of the class (e.g. Sepulveda-Rebolledo et al., 2024, [37]).

The functionality of AfFur has been determined experimentally, demonstrating that the *fur* gene can complement *fur* deficiency in *E. coli* in an iron-responsive manner [38]. Also, the DNA sequence that AfFur binds to (the *Fur* box) is present in multiple genes along the *A. ferrooxidans’* genome. These DNA recognition sequences have been validated through electrophoretic mobility shift assays (EMSA). Among them are genes related to iron acquisition (*gloA and feoB*), transport (*copB*, *abcS4*, *mntH*), other regulators (*iscR* and *phoB*), and genes codifying iron-containing proteins (*hppH*, *fdx1*) [39], [40]. Additionally, gene expression analyses have shown that the Fur regulator and many of its targets are induced in conditions of increased pH [41]. This response could be comparable to the one described for *Helicobacter pylori*, where Fur regulates the expression of genes participating in the acid shock response [42].

Despite these facts, so far there is no structural or spectroscopic information available on the Fur protein from *A. ferrooxidans* (AfFur). Considering the particularities of the niche (rich in soluble iron) and the biology of *A. ferrooxidans* (for whom iron is nutrient, energy and electron source), and given that the Fur transcription factor has a role in controlling a wide variety of processes (that require concerted signal integration)*,* we hypothesized that AfFur differs from other Fur protein described to date in its structural features. Thus, we aimed to evaluate how this protein responds to the unique iron concetrations and redox conditions of its habitat. For this we identified which amino acids are involved in iron recognition (thus forming the regulatory site), we evaluated whether it contains other cofactors, and we assessed how it is activated to exert its regulation over known target genes. AfFur, the Fur protein from *A. ferrooxidans*, is uniquely adapted to thrive in an iron-rich environment, serving as both sensor and regulator to meet the organism's exceptional needs for iron as a nutrient, energy, and electron source.

## 2 Materials and methods

### 2.1 Cloning of the *Acidithiobacillus ferroxidans* fur gene

A 529 bp fur coding region was obtained from the Acidithiobacillus ferroxidans ATCC 23270 genome amplified by PCR and cloned into a pLATE vector using the aLicator LIC cloning & expression system from Thermo Fisher Scientific (N-terminal His-tag, #K1251) according to the manufacturer’s recommendations. The resultant plasmid was named pFurAf, and all further mutants were derived from that plasmid by site-directed mutagenesis using the Phusion Site-Directed Mutagenesis Kit (Thermo Fisher Scientific). Primers were synthesized by IDT Integrated DNA Technologies (Table 1). The plasmid was transformed into *E. coli* strain DH5ɑ for long-term storage and into *E. coli* strain BL21(DE3) for protein overexpression. In all cases, clones were checked by colony PCR and DNA sequencing (Macrogen sequencing services). DNA isolation and routine manipulations were carried out following standard protocols as described by Sambrook [43] or by the manufacturers of the reagents. Plasmid DNA was prepared with the Wizard Plasmid Miniprep Kit (Promega) or the QIAprep Spin Mini-kit (Qiagen).

**Table 1:** List of oligonucleotides primers and probes used in this study.

Primers	5'-3′ Sequence	Observation
AfFur-WT-Fw	ATGATCGACGAACGAATGA	Primer forward gen AfFur
AfFur-WT-Rv	CTAATCCGTGCTGGCTCC	Primer reverse gen AfFur
AfFur-WT-pLate51-Fw	GGTGATGATGATGACAAGATGATCGACGA	pLATE cloning primer forward gen AfFur
AfFur-WT-pLate51-Rv	GGAGATGGGAAGTCATTACTAATCCGTGCT	pLATE cloning primer reverse gen AfFur
Af C96A-Fw	CATATGGTGGCGACTGCCTG	Mutagenesis primer
Af C96A-Rv	ATCGTGGTGGCCGGTTTCAT	Mutagenesis primer
Af C99A-Rv	CATATGATCATCGTGGTGGCCGG	Mutagenesis primer
Af C99A-Fw	GTGTGTACTGCCGCGGGTAAGG	Mutagenesis primer
Af C136A-Rv	ATAGAGATAGAGGCTGTGGTGGCTGATAAA	Mutagenesis primer
Af C136A-Fw	GGCACCGCGCTTGGC	Mutagenesis primer
Af C145W-Fw	GTGGGGATTTGGTCACTAAGG	Mutagenesis primer
Af C145A-Rv	GTCCTGCATGCCAAGACAGG	Mutagenesis primer
Af C145A-Fw	GTGGGGATTGCGTCACTAAGG	Mutagenesis primer
Af E84A-Rv	CTTATCGCCCTCAAAGTGGTGCCTTC	Mutagenesis primer
Af E84A-Fw	GCGGTCTTTGCGCTCAATGAAA	Mutagenesis primer
Af H91K-Rv	ATTGAGCTCAAAGACCGCCTTATC	Mutagenesis primer
Af H91K-Fw	GAAACCGGCCACAAGGATCATATGG	Mutagenesis primer
Af H91D-Rv	CATTGAGCTCAAAGACCGCCTTATCG	Mutagenesis primer
Af H91D-Fw	AAACCGGCCACGACGATCATATG	Mutagenesis primer
Af H93A-Rv	GCCGGTTTCATTGAGCTCAAAGAC	Mutagenesis primer
Af H93A-Fw	CACCACGATGCGATGGTGTG	Mutagenesis primer
Af E104K-Fw	TGGTAAGGTACTGAAATTTTTCGATGAGATGCTG	Mutagenesis primer
Bio-MntH-Fur-box	5Biosg/GGCATCAATAAACGGGAATCATTCTCGTCTACC	EMSA probe – Biotin Labelled
MntH-Fur-box	GGCATCAATAAACGGGAATCATTCTCGTCTACC	EMSA probe
MntH-Fur-box-Alex	GGCATCAATAAACGGGAATCATTCTCGTCTACC/3AlexF488N	Fluorescence probe Alexa fluor 488 labelled
Bio-Msfb-Fur-Box	5Biosg/GATGATGAATGAATAAGTTTATTATGATC	EMSA probe – Biotin Labelled
Msfb-Fur-Box	GATGATGAATGAATAAGTTTATTATGATC	EMSA probe

### 2.2 Overexpression and purification of recombinant AfFur

Using the pLATE51 vector, the recombinant AfFur protein was overexpressed, containing six additional histidine residues at the N-terminal end, which facilitated its purification through affinity chromatography. The wild-type AfFur protein and its mutants were overexpressed in E. coli strain BL21(DE3) according to the following protocol. An *E. coli* BL21(DE3) pFurAf fresh colony was grown for 14 hours at 37°C in 5 mL of Luria-Bertani (LB) medium supplemented with 100 μg/mL ampicillin. The overnight culture was transferred to 0.5 L of LB medium supplemented with 100 µg/mL ampicillin and then incubated with shaking at 37°C and 220 rpm. When the bacterial culture reached an optical density of 0.6 at 600 nm, cells were induced with 1 mM isopropyl ß-D-1-thiogalactopyranoside (IPTG) and then incubated with shaking for 14 h at 30°C and 200 rpm. Cells were harvested by centrifugation at 8000 xg and 4°C for 10 minutes, resuspended in buffer A (150 mM NaCl, 50 mM imidazole, 50 mM Tris-HCl pH 7.85, 1 mM PMSF) and then lysed by sonication. The lysate was centrifuged at 17000 xg at 4°C for 30 minutes and the supernatant was filtrated through a 0.22 µm Whatman Uniflo Syringe Filter (GE Healthcare Life Sciences).

The AfFur protein was purified by Immobilized Metal Affinity Chromatography (IMAC) In a Fast Protein Liquid Chromatography (FPLC) ÄKTA Prime Plus system using 1 mL His-Trap HP columns. The chelating group is precharged with nickel (GE Healthcare Life Sciences, Cytiva) according to the manufacturer’s recommendations. The protein was eluted with in linear gradient mode using a 500 mM imidazole in buffer A, the fractions were collected to 80 mM imidazole. Then the fractions were analysed using a Coomasie-stained SDS-PAGE and western blotting using Anti-6x His Tag Monoclonal antibodies (Invitrogen).

Protein separation was evaluated using SDS-PAGE following the method described by Laemmli (1970) [44]. A discontinuous gel system was prepared, consisting of a stacking gel (4 %) and a resolving gel (16 %). Samples were denatured by boiling at 95°C for 5 minutes in loading buffer (125 mM Tris-HCl pH 7.6, 5 % SDS, 20 % glycerol, 10 % β-mercaptoethanol, 0.01 % bromophenol blue). Electrophoresis was carried out at 150 V in running buffer (25 mM Tris, 192 mM glycine, 0.1 % SDS, pH 8.3). After electrophoresis, proteins were visualized by Coomassie Brilliant Blue staining (0.1 % Coomassie brilliant blue, 40 % methanol, 10 % acetic acid) followed by destaining (20 % ethanol, 10 % acetic acid).

The corresponding fractions were pooled and concentrated using 10 kDa Amicon Ultra 4 mL Centrifugal Filters (Merck Millipore). Finally, to remove contaminants, the concentrated fraction was loaded onto a Q Sepharose Fast Flow column (GE Healthcare Life Sciences) and then eluted with 750 mM NaCl in linear gradient mode. Fractions were pooled, concentrated in buffer S (150 mM NaCl, 50 mM Tris-HCl, 25 % glycerol, 0,5 mM DTT, pH 7.85) and then stored at −20°C for later analysis. Protein concentrations were determined by the Bradford assay using BSA as the standard [45].

### 2.3 Electrophoretic mobility shift assays (EMSA)

For AfFur DNA-binding analysis, EMSA experiments were performed using the LightShift Chemoluminiscent EMSA kit (Thermo Fisher Scientific) according to the manufacturer’s recommendations. The double-stranded DNA probe containing the well-characterized fur box of the MntH gene from A. ferroxidans was 5′-end labelled with biotin by Integrated DNA Technologies (Table 1). This protocol was performed following the indication of Quatrini et al. [38.].

Preparations of AfFur and its mutants were equilibrated in 20 µL of reaction buffer (10 mM Tris pH 7.5, 50 mM KCl, 5 mM MgCl_2_, 0.1 mM MnCl_2_, 1 mM DTT, 2.5 % glycerol, 50 ng/µL poly(dI-dC), 0.05 % NP-40). A biotin-labelled probe was added at a final amount of 20 fmol, and a 200-fold excess of non-labelled probe was added as a specificity control. Tubes were incubated for 30 minutes at room temperature and immediately mixed with loading buffer to be resolved in a non-denaturing polyacrylamide (5 % w/v) gel electrophoresis at 90 V for 90 minutes in Tris-borate-MnCl_2_ buffer (44.9 mM Tris, 44.9 mM boric acid, 0.1 mM MnCl_2_, pH 8.3) at 4°C. After electrotransfer to a nylon membrane and UV-crosslinking, retardation was examined using the Chemiluminiscent Nucleic Acid Detection kit (Thermo Scientific) according to the manufacturer’s recommendations. Its chemiluminiscent signal was detected and the image analyzed in the G:Box Chemi XRQ Gel Documentation System (Syngene, UK).

### 2.4 Generation and reconstitution of Apo-Fur

The purified Hist-tag AfFur (**Holo-Fur**) was dialyzed overnight at 4°C with 20 mM Tris-HCl pH 7,4, 1 mM DTT, 30 % glycerol. Finally, the protein was concentrated by ultrafiltration at 7500xg for 15 min at 4°C in Amicon tubes, CO 10 kDa.

To prepare a Fur protein without the metallic atom (**partially-apoFur**), the following steps were carried out. The Holo-Fur was dialyzed overnight at 4°C with 20 mM Tris-HCl pH 7,4, 1 mM DTT, 100 mM EDTA. Then it was further dialyzed with 20 mM Tris-HCl pH 7,4, 1 mM DTT, 30 % glycerol at room temperature for 3 hrs.

To obtain the protein (**ApoFur**) without its Fe-S clusters and the others cofactors (iron and zinc atom), the Holo-Fur was incubated at 95°C for 5 minutes in the presence of 100 mM EDTA and 500 mM DTT. The excess DTT and EDTA were removed using a Hitrap Desalting gel filtration column (GE Healthcare) and dissolved in 20 mM Tris-HCl (pH 7.4), 1 mM DTT, and 30 % glycerol.

For the reconstitution of the **partially-apo-Fur**, 50 µM of this preparation was incubated on ice for 20 minutes with 3 mM FeCl_3_ dissolved in sodium citrate (400 µM, pH 7.0) [13]. To remove excess iron, the protein was washed with sodium citrate buffer (400 µM, pH 7.0), through an ultrafiltration cycle in Amicon tubes.

### 2.5 UV–visible and inductively coupled plasma mass spectrometry (ICP-MS)

Protein concentration was determined, and its UV–visible spectrum was analyzed using a Synergy HTX Multimode Microplate Reader (Bio-Tek Instruments, USA). Quantification of metals was performed at Barnafi Krause Laboratory using a NexION 2000 ICP-MS PerkinElmer, calibrated according to internal standards. (Pearson correlation coefficient, 0.97674). The instrument detection limit is in the order of parts per billion (ppb). The sample consisted of 500 µL of AfFur protein, purified following the described protocol, at a 0.6 µM concentration. The sample was dialyzed to remove metals from the buffer. Then it was divided into two samples: AfFur (20 mM Tris/Acetic acid pH 7.4, 100 mM NaBr) and AfFur+EDTA (20 mM Tris/Acetic acid pH 7.4, 100 mM NaBr/1 mM EDTA).

### 2.6 X-Ray Fluorescence (XRF) and Extended X-ray Absorption Fine Structure spectroscopic (EXAFS)

An X-ray fluorescence (XRF) analysis and Extended X-ray Absorption Fine Structure (EXAFS) spectroscopic analysis were performed at the Brazilian Synchrotron Light Laboratory (LNLS) in Campinas, Brazil (project D04B-XAFS1–11037), using the XAFS2 beamline, directed by Dr. Narcizo M. Souza. A Ge-15 solid state detector was used, as it has an energetic resolution of 170 eV–5.9 keV, allowing the selected photons to be captured, excluding background signals and other noise sources. To detect metals contained in the AfFur sample, scanning was performed using the excitation energies of a variety of metals. The sample consisted of 300 µL of AfFur at a concentration of 15 mg/mL in 20 mM Tris HCl, pH 7.4, 1 mM DTT, and 25 mM EDTA. A manganese filter was used to attenuate the signal of the sample.

### 2.7 Circular dichroism

Experiments to determine secondary structure content were performed on a Jasco 1500 spectropolarimeter. The protein was dissolved at a concentration of 6.5 µM in 10 mM sodium phosphate pH 7.5. The recording was performed in a cell with a 5 mm optical path, from 190 nm to 600 nm, at an interval of 1 nm, with 3 accumulations, a speed of 50 nm/min, a bandwidth of 1 nm, all at 25 °C. Capito software [46] was used for data analysis and subsequent determination of secondary structure content.

The alignment was done with MAFFT in Geneious Prime 2024.0.7. The evolutionary history of the Fur family protein representatives was inferred by using the Maximum Likelihood method and Le 2008 model [47] as implemented in MEGA11 [48].

### 2.8 Three-dimensional model of AfFur and molecular dynamics simulations

Multiple alignments were generated using Clustal Omega [49], and their visualization and analysis was done using the Jalview [50] and EsPript 3.0 [51]. The NCBI database sequence accession number are listed in table S1 (supplementary material).

Fur protein superfamily amino acid sequences were recovered from the RefSeq repository [52] using Blastp from the BLAST suite [53]. Multiple sequence alignments were carried out using Clustal Omega. Visualization and analysis of alignments were performed using the MEGA 11 software. The template selected for the model was the Fur protein from Vibrio cholerae [15]. Sequence identity between AfFur and the template was 52 %. The 3D structure of AfFur was obtained through the MODELLER software [54] and Alphafold server [55]. Importantly, no significant difference was found between the two predictions presumably because both models are based on a number of high-resolution crystal structures.

The evaluation of the molecular models was carried out through an analysis of the Ramachandran graph [56] by the Rampage software. All models built had > 90 % of their residues in the permitted areas. The Verify3D method [57] indicated that the models are within limits allowed in their structured regions. The spatial analysis and visualization of the proteins were performed with the VMD [58] and Pymol software (The PyMOL Molecular Graphics System, Version 2.0 Schrödinger, LLC).

The selected protein model was solvated and embedded in a water box using the VMD software and Na^+^ ions to neutralize the system. Two cycles of molecular dynamics simulations were performed with the NAMD 2.13 software [59] and the CHARMM36 force field [60].The first cycle consisted of 20,000 minimization steps and 200 ps of equilibration, with a harmonic restraint of 3 kcal*mol-1 * Å-2 applied to the backbone atoms; the second cycle of 15,000 minimization steps and 200 ns of equilibration with no restraints. Both simulations were performed under periodic boundary conditions and an isobaric-isothermal setting (NPT).

Once an optimized model was obtained, the cofactors were accurately positioned within their respective sites using the AutoDock software [61]. Specifically, an iron ion was placed at site 1, a zinc ion at site 2, and a [4Fe-4S] or [2Fe-2S] cluster was positioned at site 3.

The structure of the cluster was obtained from the Protein Data Bank [62] using the "search by ligands" function. The [4Fe-4S] and [2Fe-2S] cluster was selected for further analysis being the form that is most frequently present in proteins containing iron-sulfur clusters, and given that this structure is usually coordinated by four cysteine residues [63].

Multiple types of iron-sulfur clusters were evaluated for the simulations. As the CHARMM36 force field does not include parameters for iron ions and Fe-S clusters, we had to build topology and parameter files for these cofactors. In the topology file, five new atom types were defined: two for iron and sulfur atoms from the Fe-S cluster in an oxidized state (FEO and SO), two for the same atoms in a reduced state (FER and SR), and one for Fe^2+^ ions (FEI). Atom masses were obtained from the "top_all22_prot_metals" from CHARMM36. Atomic charges were obtained from a study by [64]. Following guidelines from the same study, FE-S bonds, S-FE-S and FE-S-FE angles, and S-FE-SG-CB y FE-S-FE-SG dihedrals on the Fe-S cluster were defined. Covalent bonds between iron atoms from the cluster and sulfur atoms from the cysteines were also defined in the topology file. Parameters for all these types of bonds, angles, and dihedrals were obtained from the same study by Smith et al. [64]. The Lennard-Jones parameters were obtained from a study by De Hatten 2007 et al. [65].

In the psf file for the protein, the [4Fe-4S] and [2Fe-2S] cluster were parametrized as in the oxidized state [66]. Using VMD, the model was inserted into a TIP3P water box and sodium ions were added to neutralize the system. Using NAMD, the CHARMM36 force field, and the built parameter file, 10,000 minimization steps and 120 ns of equilibration were performed on the system, under the same conditions as the previously described simulation. To obtain the final model for AfFur, the last 1000 frames of the molecular dynamics were taken, the structures from each frame were superimposed, and an average structure was calculated. From the optimized model, we generated four AfFur mutants. Based on results from the EMSA assays, we decided to further analyze mutants H91D, H91K, C99A, and C145A through molecular dynamics. Mutations were performed using the Mutator 1.3 plugin from the VMD software. Systems for each mutant were set up and molecular dynamics simulations were performed following the same protocol described for the AfFur wild type model.

## 3 Results

### 3.1 AfFur 1ry structure exhibits unique characteristics compared to studied orthologs

**Fig. 1:**
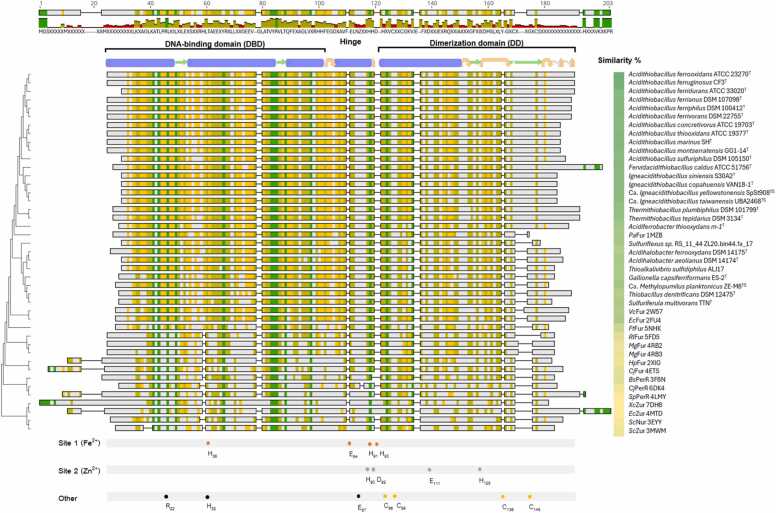
Multiple sequence alignment of AfFur orthologs of the Acidithiobacillia class and Fur proteins with known structures in PDB. The alignment was done with MAFFT in Geneious Prime 2024.0.7. The evolutionary history of the Fur family protein representatives was inferred by using the Maximum Likelihood method and Le_Gascuel_2008 model as implemented in MEGA11. The tree with the highest log likelihood (-7515.30) is shown to the left. Initial tree(s) for the heuristic search were obtained automatically by applying Neighbor-Join and BioNJ algorithms to a matrix of pairwise distances estimated using the JTT model, and then selecting the topology with superior log likelihood value. A discrete Gamma distribution was used to model evolutionary rate differences among sites (5 categories (+G, parameter = 2.4242)). The tree is drawn to scale, with branch lengths measured in the number of substitutions per site. This analysis involved 43 amino acid sequences. There were a total of 211 positions in the final dataset. Amino acid sequence similarity similarity is depicted as a schematic alignment and colored according to the sequence conservation inferred form the Blosum 62 score matrix as follows: yellow (60–80 %), ochre (80–99 %), green (100 %), with residues displaying less that 60 % conservation shown in white. Similarity values of the different orthologs/paralogs in the alignment to AfFur (ATCC 23270 T) is indicated to the right and colored as follows: yellow (<40 %), pale green (40–60 %), green (>60 %). The N-terminal DNA-binding domain (DBD), the C-terminal dimerization domain (DD) and the connecting hinge region are indicated above, along with the secondary structure prediction of AfFur. Residues conforming the co-repressor iron-binding pocket (orange circles; H36, E84, H91, H93) and those described as relevant in the coordination of the structural metal Zn^2+^ (grey circles, H90, D92, E111, H128) are indicated below, following AFFur´s notation (residues position). Additional residues analyzed in this work are pinpointed as other. PDB accession numbers are indicated in the figure margin and proteins sequence identifiers are listed bellow: ACK78219 (ATCC23270), WP_215877620 (CF3), MBU2715368 (ATCC 33020), WP_163095519 (DSM 107098), WP_271779603 (DSM 100412), WP_014030069 (DSM 22755), MBU2737344 (ATCC 19703), ACI62939 (ATCC 19377), WP_101536577 (SH), WP_215843000 (GG1–14), WP_123103429 (DSM 105150), MCY0871381 (ATCC 51756), WP_248884703 (S30A2), WP_226827788 (VAN18–1), HGE68344.1 (SpSt908_MAG), WP_312261756 (UBA2468), WP_341370352 (TPL), WP_211218724 (TTP), WP_083996317 (m-1), MCK4743299 (RS_11_44 ZL20.bin44.fa_17), WP_076835706 (DSM 14175), WP_070074078 (DSM 14174), WP_026289749 (ALJ17), WP_013292836 (ES-2), CAH7811198 (ZE-M8), WP_018077389 (DSM 12475), WP_124703505 (TTN).

The AfFur protein is composed of 158 amino acids and has a molecular weight of 17,9 kDa. Considering the additional amino acids added due to the expression vector, its size is 22 kDa, which was confirmed by mass spectrometry (Figure S1). AfFur is globally conserved between members of the *Acidithiobacillia* class, with amino acidic sequence identity levels above 65 % (Table S3 and S4). It also shares between 18 % and 53 % sequence identity with other members of the superfamily for which crystals structures are available (Table S3), showing the highest identity to the Fur ortholog from *Pseudomonas aeruginosa* (53.6 %) and *Vibrio cholerae* (52.0 %). The sequence alignment in Fig. 1 shows the general conservation of the proteins´ amino acidic sequence and its predicted secondary structure topology, which is typical of members of the Fur superfamily consisting of an N-terminal DNA-binding domain (DBD) and a C-terminal dimerization domain (DD) connected by a hinge region. The DBD of AfFur is composed of four α-helices (H1: Ser8 - Leu16; H2: Thr19 - Ile28; H3: Thr38 - Thr49; H4: Leu55 - Gly69), while the DD consists of an α-helix (Glu108 - Arg121) and a β-sheet (Gly122 - Leu137), both displaying sequence identity levels above 80 %. Residues described in these protein orthologs as relevant in the interaction with DNA (Arg22), the coordination of the structural metal Zn^2+^ (PaFur: His32, Glu80, His89 and Glu100 [67] and those conforming the co-repressor iron-binding pocket (PaFur: His86, Asp88, Glu107, His124) are present in the *A. ferrooxidans* ortholog (and in other class members), supporting general conservation of function. Despite these similarities, AfFur and its orthologs within the class *Acidithiobacillia* exhibit differences from other structurally characterized Fur family regulators in the configuration of the C-terminal CX_n_C motif, which displays a spacing (CX_8_C). While the precise function of these cysteine residues remains unclear. It is estimated that the redox state of these cysteines and the coordination of zinc are crucial for stabilizing EcFur in its dimeric form, as demonstrated by D’Autréauxet al. (2007) [68].

This is supported by evidence from related Fur family proteins, as discussed by Pinochet-Barros and Helmann (2018) [94.]. For EcFur, mutagenesis studies revealed that the residues Cys-93, Cys-96, and Cys-133 are essential for the [2Fe-2S] cluster to bind to the protein [11].

### 3.2 AfFur reconstructed dimer unveils three potential ligand-binding sites

**Fig. 2:**
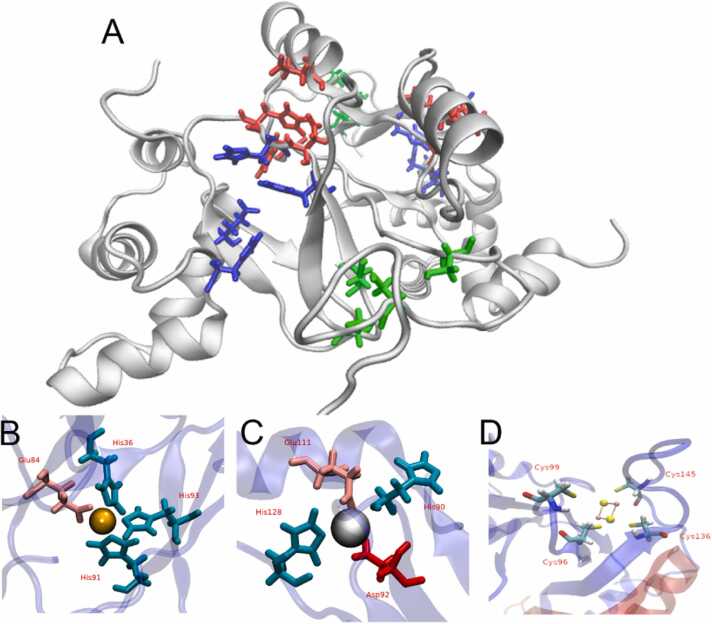
Model of the predicted structure of the AfFur protein dimer and metal coordination sites. (A)The diagram shows the 3D model of the AfFur dimer. In blue the site 1, red site 2 and green site 3. (B) Site 1:_structural metal binding site 1 (His36, Glu84, His91, His93). (C) Site 2:_corepressor binding site 2 (His90, Asp92, Glu111, His128). (D) Site 3: Fe-S cluster coordination site (Cys96, Cys99, Cys136, Cys145). The co-repressor (iron, orange) and the structural metal (Zn^2+^, grey) are drawn as spheres. The square structure in (D) represents the [2Fe-2S] cluster, with sulfur atoms in yellow and iron atoms in pink. Metal-binding site residues are shown in licorice representation.

Given the conserved and differential characteristics observed in the primary sequence of AfFur, a 3D model of the protein was built using comparative modelling and structure optimization procedures. The resulting model for the monomer and the reconstructed dimer obtained by structural superposition of the AfFur monomer and the PaFur dimer is shown in Fig. 2**A**. SDS-PAGE electrophoresis of the purified protein (Figure S2) and western blot assays (Figure S3), along with size-exclusion chromatography (data not shown), confirmed that AfFur exists in a dimeric state in solution.

Further analysis of the 3D model confirmed the conservation and identical spatial arrangement of the residues involved in corepressor and structural metal coordination. Site 1 is potentially conformed by His36, Glu84, His91 and His93 (Fig. 2**B**, site 1); all of these residues are known to belong to the regulatory site of PaFur, VcFur, FtFur, CjFur, MgFur, RlMur and HpFur [69] and create an tetrahedral coordination sphere for the iron atom, located entirely within the protein's dimerization domain. Predicted site 2 is ligated by His90, Asp92, Glu111 and His128 (Fig. 2**C**, site 2); all of these residues are known to be part of the structural site of PaFur, VcFur, CjFur, MgFur, RlMur and HpFur [69]. The regular tetrahedral environment generated by the side chains of these four residues positions the Zn^2+^ atom between the monomer domains, and is consistent with the structural role attributed to this atom. The third site emerging from the AfFur 3D model (Fig. 2**D**, site 3) is one composed of four cysteines belonging to two paired CX_n_C motifs; the CX_2_C motif (Cys96 and Cys99) and the CX_8_C motif (Cys136 and Cys145). These residues are partially equivalent to the ones known to make up the second structural site of FtFur, CjFur, MtZur, BsPerR, ScZur, CjPerR, SpPerR, HpFur and EcFur.

Given this background and the inferred characteristics of site 3 (a coordination environment consisting of 4 cysteines and a large predicted pocket size) we evaluated the capacity of the AfFur dimer model to bind a larger sized Fe-S cluster. Results obtained via Molecular Dynamics (MD) and Energy Minimization (ME) routines indicated that an [2Fe-2S] or [4Fe-4S] cluster could indeed be accommodated in site 3 of AfFur and likely also in other orthologs of Fur from known members of the *Acidithiobacillia* class.

### 3.3 AfFur ligand-binding sites coordinate Fe^2+^, Zn^2+^ and the Fe/S cluster

To determine which transition metal ions reside in each of the binding sites of AfFur, we combined bioinformatic analysis of metal-binding proteins that have known crystal structures and spectroscopic analysis of AfFur protein preparations.

**Fig. 3:**
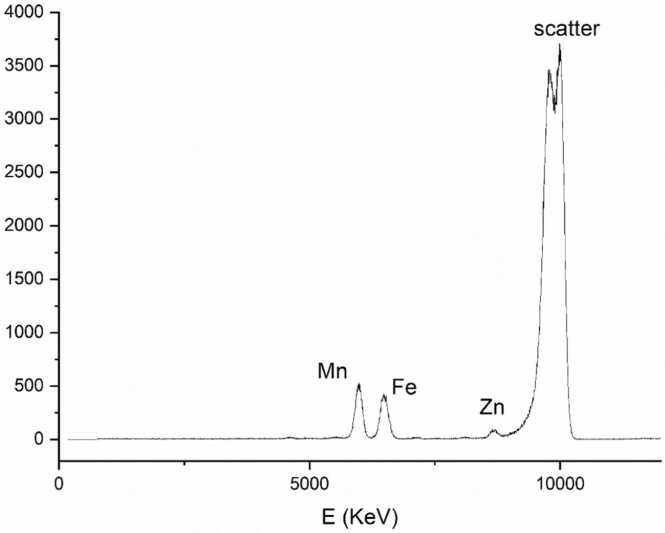
Absorption spectrum detected for AfFur, showing metallic ions present in the protein. X-axis: energy (KeV). Y-axis: counts. Peaks indicate the presence of iron and zinc atoms. The Mn peak corresponds to the attenuation filter used in the detector. The scatter peak corresponds to the electron beam used to excite the sample.

First, we studied the metalloprotein structures available on the Protein Data Bank as of May 2023. Analyses performed with the software AFAL [70] and GSP4PDB [71] showed that His, Asp Glu and Cys are the residues most often coordinating Zn^2+^ (Figure S4 **A**) and Fe^2+^ (Figure S4 **B**) atoms in the set of 21.159 proteins analyzed. These residues conform to the configuration of predicted site 2 in the *A. ferrooxidans* orthologs 3D structural model (AfFur-site 2: His90, Asp92, Glu111 and His128), and to the site inferred to be the structural-metal binding pocket in characterized Fur orthologs (Table 2). The presence of both metal atoms in AfFur protein samples could be confirmed by X-Ray Fluorescence (XRF, Fig. 3) and inductively coupled plasma-mass spectrometry targeting multiple metals (ICP-MS, Table 3).

**Table 2:** Amino acidic composition of metal-binding sites of Fur superfamily proteins of known structure. the X represents any residue between the amino acid.

**Site N.º**	**Type** **of site**	**Amino acids** **involved**	**Metal ion**	**Proteins (PDB ID) containing** **the site**
**Site 1**	regulatory	His, Glu, HisXHis, Glu	Zn(II)	*Hp*Fur (2XIG)
			Fe(II)	*Ft*Fur (5NHK)
			Mg(II)	*Mg*Fur (4RAZ)
		His, Glu, His, Glu	Zn(II)	*Pa*Fur (1MZB)
		His, Glu, HisXHis	Zn(II)	*Vc*Fur (2W57), *Rl*Mur (5FD6)
		His, AspX5HisXHisX10 Asp	Mn(II)	*Bs*PerR (3F8N), *Cj*PerR (6DK4)
		His, AspX5HisXHis	Zn(II)	*Li*PerR (5NL9)
		Asp, Cys, HisXHis	Zn(II)	*Mt*Zur (2O03), *Sc*Zur (3MWM)
		His, Cys, His, Glu	Zn(II)	*Ec*Zur (4MTD)
		Glu, HisXHisXHis, Glu	Fe(II)	*Cj*Fur (6D57)
		His, HisXHisXHis	Ni(II)	*Sc*Nur (3EYY)
		HisXHis, Asn, His, HisXHis	Ni(II)	*Sp*PerR (4I7H)
**Site 2**	structural	HisXAsp, Glu, His	Zn(II)	*Pa*Fur (1MZB), *Hp*Fur (2XIG), *Vc*Fur (2W57), *Rl*Mur (5FD6)
		Asp, Glu, His, 2 H2O	Zn(II)	*Cj*Fur (6D57)
		HisXAsp, GluXXGln, His	Mn(II)	*Mg*Fur (4RAZ)
		HisXHis, Glu, His	Zn(II)	*Mt*Zur (2O03), *Sc*Zur (3MWM)
		HisXHis, His, 3 H2O	Ni(II)	*Sc*Nur (3EYY)
**Site 3**		CysXXCys, CysXXCys	Zn(II)	*Hp*Fur (2XIG), *Cj*Fur (6D57), *Ft*Fur (5NHK), *Mt*Zur (2O03), *Sc*Zur (3MWM), *Ec*Zur (4MTD), *Bs*PerR (3F8N), *Sp*PerR (4I7H), *Cj*PerR (6DK4)

**Table 3:** ICP-MS quantification of metals bound to AfFur, mutant and AfFur treated with EDTA.

	Protein concentration	Fe (ppb)	Zn (ppb)
Holo Fur N° 1	2 µM	201.9	41.03
Holo Fur N° 2	2 µM	195.7	41.61
Holo Fur N° 3	2 µM	198	41.51
x		198.5	41.38
s		3.112	0.311
%RSD		1.568	0.752
uM		3.5446	0.627
	Protein concentration	Fe (ppb)	Zn (ppb)
partially ApoFur - N°1	2 µM	153.1	6.955
partially ApoFur - N°2	2 µM	157.1	7.174
partially ApoFur - N°3	2 µM	156.7	7.316
x		155.6	7.148
s		2.206	0.182
%RSD		1.417	2.542
uM		2.7786	0.1083
	Protein concentration (mg/mL)	Fe 56 Helium KED (µg/L)	Zn 66 Helium KED (µg/dL)
Holo Fur	0.705	1371.16	181.67
Apo Fur	0.203	−2.83	1.48
H91D	0.451	772.37	119.57
C96A	0.174	−6.75	10.92
control: taq polymerase	-	−16.56	1.54
control: lysozyme	-	−16.17	1.00

*The molecular weight of recombinant AfFur is 21776 Da per monomer.

**Fig. 4:**
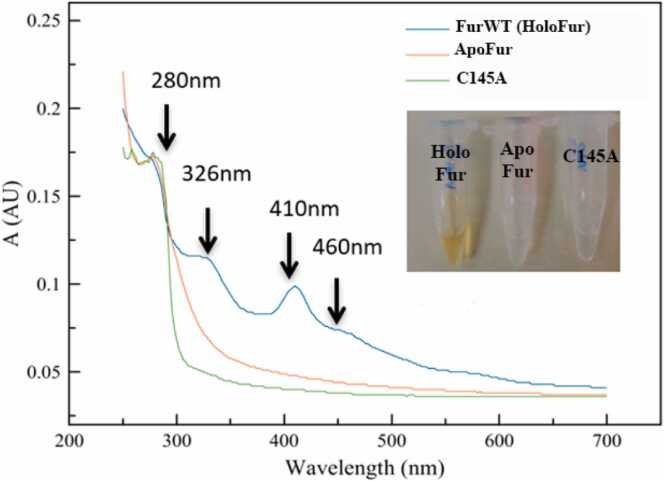
UV–visible spectra for wild-type AfFur (**HoloFur**), C145A mutant **(C145A**) and Fur without the cofactors (**ApoFur**). Wild-type Fur displayed the characteristic UV–visible absorption spectra for iron-sulfur proteins, with a peak around 410 nm (arrow) as well the typical brown color of iron-sulfur proteins. This does not happen to C145A mutant or the Apo Fur.

Profiling of amino acids in coordination environments consisting of four cysteines (like site 3 from AfFur) showed that the most common ligands for this type of site are iron-sulfur clusters (Figure S4 **C**), which is consistent with descriptions for other metalloproteins in literature [72], [73], [74], [75], [76], [77]. During AfFur protein purification, brownish-colored fractions were consistently obtained (Fig. 4), a phenomenon that has been described frequently for proteins containing iron-sulfur clusters [75]. In addition, the UV–visible spectroscopy analysis displayed peaks at 280 nm, 326 nm, 410 nm and 460 nm (Fig. 4), which is also characteristic of proteins with [2Fe-2S], [3Fe-4S] and [4Fe-4S] clusters [11], [63], [78], [79]. In addition, dialysis of the protein using an EDTA buffer caused it to lose iron and zinc (Table 3). These results strongly support the presence of a structural Zn site combined with an iron-sulfur cluster, further underscoring AfFur's adaptation to its unique iron-rich environment.

**EXAFS analysis support AfFur site 3 as an Fe-S cluster binding pocket**

**Fig. 5:**
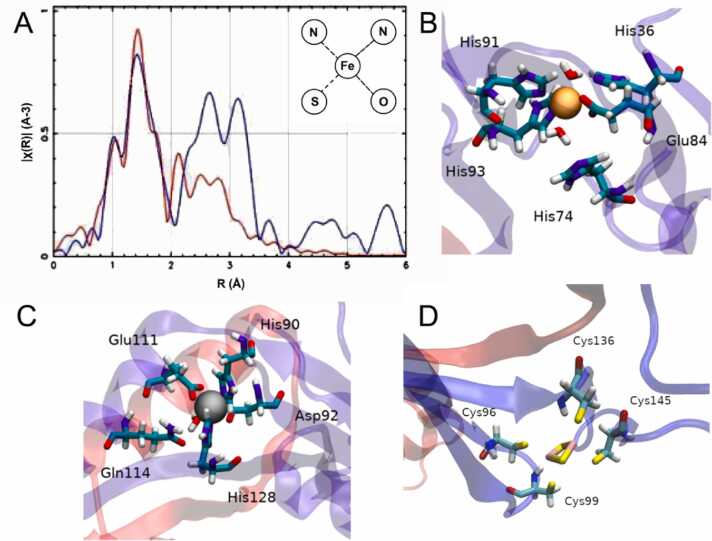
**Characterization of the ligand-binding microenvironments of AfFur.** (**A**) Fourier-transformed EXAFS spectrum of iron. The experimental data is depicted in blue, while the red line illustrates the best fit derived from the theoretical EXAFS calculations based on the proposed atomic model of the microenvironment The inset depicts the proposed atomic model of the microenvironment, consisting of two nitrogen atoms, one oxygen atom, and one sulfur atom. The radial distances were measured in angstroms. Molecular dynamics of (**B**) Site 1 containing an Fe^+2^ ion (brown) coordinated by amino acids Glu84, His91, His93 and two water molecules, (**C**) Site 2 containing a Zn^+2^ ion (gray) coordinated by His90, Asp92, Glu111, His128 and a water molecule and (**D**) Site 3 containing a [2Fe-2S] cluster coordinated by Cys96, Cys99, Cys136 and Cys145.

To characterize the interaction of the iron atoms with AfFur, we next used Extended X-ray Absorption Fine Structure spectroscopy (EXAFS) and analyzed the atomic coordination surrounding the iron atoms. The EXAFS spectrum obtained for iron is displayed in (Figure S5). and its Fourier Transform (FT) is shown in Fig. 5**A**. An asymmetric peak of between 1 and 2 Å was observed in the radial distance graph for the first coordination shell (Fig. 5**A**), which indicates the presence of multiple types of nearby atoms. Furthermore, the distinct splitting of the EXAFS peak at 4.6 Å depicted in Figure S5, is a typical indicator of the presence of histidine amino acid residues in the metal's environment as reported by Stranger et al. [80.] and Lucarelli et al. [80], [81]. The nature of the iron microenvironment was further evaluated through multiple atomic coordination models, considering tetrahedral, octahedral, and planar geometries using various combinations of oxygen, nitrogen, sulfur, and iron atoms. For each proposed model, the theoretical EXAFS spectrum was calculated using the IFEFFIT software [82], and these were subsequently compared with the experimental spectra. The optimal model to the experimental data is shown as a red line in Fig. 5**A**. This model comprises one sulfur atom, one oxygen atom, and two nitrogen atoms, which according to the literature occur in metalloproteins featuring environments containing histidine’s and glutamates, such as in the case of MtZur [81]. Despite this being a possible scenario for AfFur (as His36, His91, Glu84 are conserved in the sequence alignment and 3D model), the spectrum obtained revealed a peak at 2 Å (Figure S5) that suggests a different type of interaction compared to that reported for MtZur. Alternatively, the EXAFS spectrum obtained could be capturing signals from various iron atoms, each embedded in a unique molecular environment, which would complicate the accurate simulation of the iron coordination environment. The simulation that fitted best the experimental curve support a coordination sphere for iron characterized by the presence of two nitrogen atoms (His), one oxygen atom (Glu or Asp), and one sulfur atom (either from a cysteine or an iron-sulfur cluster). Based on these results and the evidence gathered above we propose that Site 1 of AfFur contains an Fe^2+^ ion and Site 2 a Zn^2+^ ion. The nature of the coordination residues and the results of the spectroscopy analysis further suggest that site 3 contains an iron-sulfur cluster likely to be an [2Fe-2S], [3Fe-4S] or [4Fe-4S] cluster. This is consistent with recent reports in a number of orthologs of Fur (VcFur and HpFur [83] and EcFur [11]) for which spectroscopic data support the presence of [2Fe-2S] clusters acting as sensors of iron during the iron homeostasis response.

In addition, experiments using EPR were conducted, but the results were inconclusive, likely due to the variety of iron types present in AfFur, which led to the overlap of multiple signals. Alternative techniques, such as Mössbauer spectroscopy, are not currently accessible to our research group or close collaborators.

These interactions between the AfFur protein putative metal-binding sites and the inferred site-specific ligands were simulated using molecular dynamics. During the simulation the metal cofactors remained stable in their respective binding sites (Fig. 5**B-D**). Amino acids within a 3 Å radius of the metal ions were considered as contributors to their coordination. For site 1 some alternations were observed between water molecules and the residue His36 in the coordination with the iron atom. The Zn^2+^ ion on Site 2 is coordinated mostly by His90, Asp92, Glu111 and His128 throughout the simulation. In certain simulation steps some of these residues alternated the coordination of the Zn^2+^ ion with a water molecule and/or with residue Gln114, which is also highly conserved.

The interaction between the protein and two types of iron-sulfur clusters (**2Fe-2S** and **4Fe-4S**) was simulated using molecular dynamics. In both simulations, the interaction remained stable (Figures S10, S13, S14). The cluster on site 3 was stably coordinated by residues Cys96, Cys99, Cys136 and Cys145 for the duration of the simulation.

### 3.4 AfFur site 1 and site 3 mutations impair metal cofactor binding and function

To experimentally confirm the role of the amino acids inferred as relevant in the interaction with the metallic cofactors, we constructed a series of mutants for site 1 (Fe^2+^) and site 3 (iron-sulfur cluster) residues and tested their ability to bind a native Fur box (*mntH*Af gene Fur box, [38], [40] through EMSA assays. Mobility shifts were contrasted against the wild type protein, in the presence or absence of competing excess unlabelled probe DNA (Table 4). Among site 1 mutations, only the H93A mutant protein lost its DNA-specific binding ability to the tested Fur box, suggesting that point mutations in the Fe^2+^ coordination are tolerable depending on the specific position (Table 4**,** Figure S6, S7). Molecular simulations for H91A H91D and H91K mutants provided insight into why these alterations did not result in a loss of functionality. Root mean square deviation (RMSD) calculations did not reveal any major differences between the secondary and tertiary structures obtained at the end of the MD simulation for wild type AfFur and those of the mutants, with the highest RMSD value being that of the H91D (2.50 Å, Figure S10, S11, S12). For the H91D mutant, the Asp91 residue adopted the metal coordinating role of His91 from the wild-type protein. In contrast, for the H91K mutant, the simulation revealed a shift in the coordination of iron. Alternative coordination was also observed involving residues His74, Glu84, His93, and three water molecules, as detailed in Table S2, suggesting site 1 is a robust and stable microenvironment for iron binding. Site 1 His 93 was the single residue involved in iron coordination in all alternative microenvironments calculated, suggesting it plays a central role in iron coordination in AfFur.

**Table 4:** Shift in electrophoretic mobility of *mntH*_*Af*_ Fur box for AfFur and mutant proteins.

	**Name**	**Labelled probe**	**Labelled probe** **+ Cold probe**	**Labelled probe** **+ EDTA**	**Number of figure**
AfFur	Holo Fur	**+**	**-**	-	S6 - A |S7 - A| | S8
AfFur	Partially apo Fur	**-**	**-**	nd	S6 - C
AfFur	HoloFur-R	**+**	**-**	nd	S6 - C
AfFur	HoloFur-R2	**+**	**-**	nd	S6 - C
Site 1	E84A	**+**	**+**	nd	S7 - A
Site 1	H91K	**+**	**+**	-	S8
Site 1	H91D	**+**	**-**	nd	S7 - B
Site 1	H93A	**-**	**-**	nd	S7 - A
Site 1	E104K	**+**	**-**	nd	S7 - A
Site 3	C96A	**-**	**-**	-	S6 - A
Site 3	C99A	**-**	**-**	-	S6 - B
Site 3	C136A	**-**	**-**	-	S6 – B | S7 - B
Site 3	C145A	**-**	**-**	-	S7 - B
Site 3	C145W	**-**	**-**	-	S6 - A

Labelled probe: probe DNA, 5’biotin-Fur box

Cold probe: is the Fur Box without biotin, unlabelled probe of DNA.

+ : the shift occurs

-: shift does not occur

+ -: the shift occurs partially

nd: not determined

HoloFur: is the Fur wildtype with the cofactors

Partially apoFur: is the FurWT without the atomic metalls

HoloFur-R: is the FurWT without the iron atom, subsequently reconstituted with Fe^2+^

HoloFur-R2: is the same sample with one additional step for remove excess of iron.

**Fig. 6:**
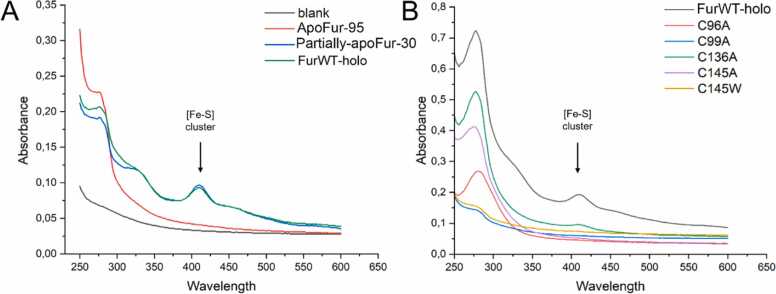
**Iron-sulfur cluster binding ability of wild type AfFur and protein variants.** (**A**) UV–visible spectra for wild type holoenzyme with its native metal cofactors (**FurWT-holo**) and the apoenzyme obtained by treating AfFur with EDTA and DTT and incubating the reaction at 95°C (**ApoFur-95**) or at 30°C (**Partially-apoFur-30**). (**B**) UV–visible spectra for AfFur wild type (FurWT-holo) and mutants C96A, C99A, C136A, C145A and C145W.

In contrast, all mutations in site 3 caused the protein to lose its ability to bind to the Fur box (Table 4, Figures S7, S8), clearly evidencing the importance of all residues in this site for the protein´s function. Circular dichroism analyses confirmed that the secondary structure of the protein is unaffected by the mutation of Cys99 (Figure S8), as did RMSD calculations (Figure S10), suggesting that structural changes in the protein are not the cause in the change of this characteristics. UV–visible spectroscopy analysis of the site 3 mutants showed that all these protein variants lost the absorption peak observed in the wild type protein at 410 nm, presumed to correspond to the iron-sulfur cluster fitting the site 3 pocket (Fig. 6**A**). Furthermore, purified protein solutions for mutants C99A and C145A lacked the brownish coloration characteristic of wild type AfFur (Fig. 4), and other proteins with Fe-S clusters, indicating the likely loss of this cofactor.

In order to confirm this interpretation EMSA assays (Figure S6, S7, S8) and absorption spectra analyses were performed on wild type AfFur protein preparations chemically treated to deplete the protein from its cofactors under harsh condition (EDTA + DTT at 95°C) to obtain the ApoFur or mild conditions (EDTA + DTT at room temperature) to obtain partially apo-Fur, and in the metal reconstituted from the partially apo-fur. Treating the protein with EDTA and DTT at 95°C caused AfFur to lose its ability to bind to the *mntH* Fur box (Table 4**,** Figure S7), along with the loss of the 410 nm UV–visible absorption peak (Fig. 6**B**), supporting that the loss of the Fe-S cluster cofactor parallels the loss of function (DNA-binding capacity; Table 4**,** Figure S7, S8). Treatment at room temperature resulted in only a partial loss of function and a partial decrease in the absorption peak (Table 4**,** Fig. 6**A, Partially-apoFur30**). The Fur box binding capacity of mildly treated AfFur was restored upon reconstitution of the partially apo-fur with Fe^3+^ (Table 4**,** Figure S6C). Therefore, we can conclude AfFur has the ability to bind an Fe-S cluster at site 3 and this factor is required for the protein to bind Fur target sequences. The molecular dynamics simulations for site 3 mutants C99A and C145A, showed that these AfFur variants cannot hold the Fe-S cluster together, and that the cluster moved away 200 Å from the site. These results complement those observed through EMSA and spectroscopy assays and further reinforce the relevance of the cysteines in the coordination of the iron-sulfur cluster in the Fur ortholog of *A. ferrooxidans*.

## 4 Discussion

This study elucidates the intricate coordination of metallic cofactors by the Ferric Uptake Regulator from *A. ferrooxidans*, offering insights into the molecular underpinnings that govern its function. By integrating bioinformatic predictions with extensive MD simulations and experimental validations, we have confirmed the presence of two distinct metal-binding sites, each playing a crucial role in AfFur protein's regulatory capabilities.

Sequence and structural alignments showed that AfFur shares a high degree of structural and functional conservation with other well characterized Fur family members, such as those from *V. cholerae* [15] and *P. aeruginosa*.

We propose that Site 1 functions as the regulatory site, while Site 2 plays a role in maintaining the structural integrity of the protein. The regulatory site is predicted to bind an iron atom, whereas the structural site is likely to coordinate a zinc atom.

The regulatory and structural metal-binding sites in the AfFur ortholog provide conserved metal-coordination, afforded by globally conserved site 1 residues His, Glu, His, His and site 2 His, Asp, Glu, His [84]. The presence of both iron and zinc as metallic cofactors in AfFur was experimentally confirmed by spectroscopy analyses. Also, EXAFS spectrometry and simulations best matching the spectral data, indicated that the iron atoms of AfFur are likely coordinated by residues of Asp, Glu, His, and Cys. Altogether, these results indicate that the AfFur regulator conforms to the functional characteristics of most of its orthologs, in agreement with previous molecular biology studies [40].

However, the AfFur regulator exhibits a distinctive configuration at the C-terminal end of its primary sequence. This arrangement facilitates the formation of a third metal-binding site, named here as site 3, similar to what has been observed in other orthologs, Zur [85] [86] and PerR [87] [88]. AfFur site 3 is characterized by the presence of four cysteine residues (Cys96, Cys99, Cys136, and Cys145), all fully conserved in other orthologs from the *Acidithiobacillia* class, and sharing a configuration that is indicative of its potential for metal-binding.

The four cysteines (Cys-93, Cys-96, Cys-133, and Cys-138) in *E. coli* Fur are highly conserved across the Fur family (Figure S15). In the work of Butcher et al. [97] , cysteine residues play a significant role in the coordination of metal ions within the structure of the Fur protein from *C. jejuni*. This site contains a zinc ion coordinated by two pairs of cysteine residues (Cys105/108 and Cys145/148). The tetracoordination provided by these cysteine pairs forms a zinc-finger motif, which is critical for maintaining the structural integrity of the protein and facilitating dimerization. This arrangement is also observed in homologous Fur proteins from other species, such as *H. pylori*. Fur protein in *F. tularensis* binds a zinc ion coordinated by four cysteine residues (Cys93, Cys96, Cys133, and Cys136). These cysteines form two pairs and are responsible for stabilizing the structure of the protein, contributing to the stability of the protein in its tetrameric form [89].

Our functional analyses of site-directed mutants provided significant insights into the metal-binding dynamics of AfFur. The mutation H93A in site 1, crucial for iron binding, resulted in a loss of DNA-binding ability, highlighting its pivotal role in iron coordination for effective gene regulation. In contrast, other tested and simulated mutations of site 1 residues showed that AfFur can tolerate certain alterations without losing functionality, likely due to compensatory interactions within the protein’s metal-binding microenvironment.

Conversely, site directed mutagenesis of site 3 residues, specifically any of the cysteines presumed to coordinate the Fe-S cluster, led to significant alterations in the protein’s properties: (a) its ability to bind DNA as evidenced by electrophoretic mobility shift assays, (b) the coloration of the protein extracts upon purification, (c) the alterations in its absorption spectra. The changes in these characteristic can be attributed to the loss of the Fe-S cluster bound to AfFur´s site 3, with little apparent effect on the overall conformation of the protein.

Fontenot et al. [11] propose that the EcFur binding of the [2Fe-2S] cluster induces a conformational change in the Fur protein in response to elevated intracellular iron levels. This conformational shift transforms Fur from an inactive state to an active [2Fe-2S]-bound repressor, enabling it to effectively regulate gene expression in *E. coli*. When intracellular free iron levels increase, Fur binds reversibly to a [2Fe-2S] cluster through conserved cysteine residues (Cys-93, Cys-96, Cys-113 and Cys 138) in *E. coli* cells [12.]. The conclusion reached by Fontenot et al. suggests that Fur senses intracellular free iron content through the binding of a [2Fe-2S] cluster, offering a novel perspective on the physiological connection between intracellular iron homeostasis and iron-sulfur cluster biogenesis. The use of an iron-sulfur cluster to monitor intracellular free iron levels is not without precedent.

In the case of regulatory proteins containing Fe-S clusters, the loss of an iron atom or alterations in the oxidation state of these atoms enable them to detect redox changes in the environment. This mechanism is observed in oxygen sensors such as *E. coli* Fnr [90], *Staphylococcus* NreB [91], *B. subtilis* Fnr [63], and the nitric oxide sensor NsrR [92], allowing them to function effectively as sensors [93]. For the Fur superfamily, we found multiple pieces of evidence demonstrating the functionality of this protein in regulating processes associated with oxidative stress [94]. Additionally, we can observe how changes in the redox state of cofactors associated with the Fur protein and its orthologs trigger either activation or repression of its regulation. In *Clostridioides difficile*, Fur exhibits redox-driven regulatory properties, with thiol-based oxidation affecting its DNA-binding activity [95]. Another example is the case of PerR, which requires H_2_O_2_ to oxidize the His residues at the iron binding site in order to apply its regulatory effect. This prevents the metal binding to the protein, resulting in the inability to bind to DNA [96].

Our findings demonstrate an association of the Fe-S cluster with site 3 of the native AfFur protein, as evidenced by its resistance to disassociation during EDTA treatment, a fact corroborated by our spectroscopic analyses. To release this cofactor, the protein had to undergo a denaturing process (heating of AfFur to 95°C for five minutes in the presence of EDTA), indicating the substantial stability of the Fe-S cluster within its native conformation. The solubility of AfFur was also affected by the loss of the Fe-S cluster, being much lower for the cysteine mutants and the apo-sate of AfFur, than for the holoprotein. In fact, the purification yield of these mutants was 20 % of that of wild-type AfFur (0.2 mg/mL vs. 1 mg/mL). This may mean that a great part of the protein becomes aggregated or degraded by the action of proteases upon loss of the Fe-S cluster. Because of this, we propose that site 3 binds an iron-sulfur cluster that has a stabilizing role in the structure of Holo-Fur. It is also possible that the cluster acts as a redox stabilizer against oxidation of these cysteines in the highly oxidant conditions of *A. ferrooxidans* environment.

Whereas [2Fe-2S] clusters are commonly found in a range of proteins where they play a role in electron transport, proteins containing [4/3Fe-4S] clusters are often involved in redox reactions where cluster transformation is a functional requirement, thus inherently designed to facilitate cluster changes under stress conditions, including acidity. Conversions between different cluster types caused by fluctuations in pH could thus an act as a regulatory signal that links pH and Fe regulatory responses in *A. ferrooxidans.* This adaptation might be an evolutionary response to the iron-rich, extremely acidic and oxidatively challenging environment that *A. ferrooxidans* inhabits, suggesting a specialized mechanism for managing iron overload and preventing oxidative stress and coordinating these responses to the acidity of the medium.

## 5 Conclusion

This study on the Ferric Uptake Regulator from *Acidithiobacillus ferrooxidans* elucidated the structure and function of its metal-binding sites, confirming the presence of three distinct sites. These include two sites that coordinate iron and zinc, typical of Fur proteins, and a third site that binds an iron-sulfur cluster. Spectroscopic analysis supported the stable binding of this cluster, which is not disrupted even in the presence of EDTA, unless the protein is subjected to high temperatures. Site-directed mutagenesis experiments revealed the critical role of specific cysteines at the third site in coordinating the Fe-S cluster and the overall functionality of AfFur, particularly its DNA-binding capability. The structural insights gained herein for AfFur highlight the evolutionary adaptations of this global regulator to the ecological niche of *A. ferrooxidans* (involving high metal concentrations, pH fluctuations and oxidative conditions) and invite further exploration into the regulatory mechanisms of metal uptake in extremophiles under changing pH condition.

## Funding

M. Arenas-Salinas was supported by the Comisión Nacional de Investigación Científica y Tecnológica (under Grant FONDECYT de Iniciación 11180665), Santander travel program , Laboratorio Nacional Luz Sincrotrón. Brasil (D04B-XAFS1-11037) and partially supported by the supercomputing infrastructure of the NLHPC (CCSS210001). This work was supported by the Agencia Nacional de Investigación y Desarrollo (ANID) under Grants FONDECYT 1221035 (R.Q.), Centro Ciencia & Vida, FB210008, Financiamiento Basal para Centros Científicos y Tecnológicos de Excelencia (R.Q.).

### Declaration of Generative AI and AI-assisted technologies in the writing process

During the preparation of this work the author(s) used ChatGPT in order to to improve language and readability. After using this tool/service, the author(s) reviewed and edited the content as needed and take(s) full responsibility for the content of the publication.

## CRediT authorship contribution statement

**Arenas-Salinas Mauricio:** Writing – review & editing, Writing – original draft, Methodology, Investigation, Funding acquisition, Formal analysis. **Quatrini Raquel:** Writing – review & editing, Writing – original draft, Methodology, Investigation. **Pohl Ehmke:** Writing – original draft, Methodology, Investigation. **Imas Francisco:** Investigation. **Obando Patricia:** Methodology, Investigation. **Olivos Andrea:** Writing – original draft, Methodology, Investigation. **Argandoña Yerko:** Methodology, Investigation.

## References

[1] Ernst J.F., Bennett R.L., Rothfield L.I. (1978). Constitutive expression of the iron-enterochelin and ferrichrome uptake systems in a mutant strain of Salmonella typhimurium. J Bacteriol.

[2] McHugh J.P., Rodríguez-Quiñones F., Abdul-Tehrani H., Svistunenko D.A., Poole R.K., Cooper C.E., Andrews S.C. (2003). Global iron-dependent gene regulation in Escherichia coli: a new mechanism for iron homeostasis. J Biol Chem.

[3] Seo S.W., Kim D., Latif H., O’Brien E.J., Szubin R., Palsson B.O. (2014). Deciphering fur transcriptional regulatory network highlights its complex role beyond iron metabolism in Escherichia coli. Nat Commun.

[4] Banerjee R., Weisenhorn E., Schwartz K.J., Myers K.S., Glasner J.D., Perna N.T., Kiley P.J. (2020). Tailoring a global iron regulon to a uropathogen. mBio.

[5] Baichoo N., Helmann J.D. (2002). Recognition of DNA by Fur: a reinterpretation of the fur box consensus sequence. J Bacteriol.

[6] Cornelis P., Matthijs S., Van Oeffelen L. (2009). Iron uptake regulation in Pseudomonas aeruginosa. Biometals: Int J role Met ions Biol, Biochem, Med.

[7] Delany I., Rappuoli R., Scarlato V. (2004). Fur functions as an activator and as a repressor of putative virulence genes in Neisseria meningitidis. Mol Microbiol.

[8] Rudolph G., Hennecke H., Fischer H.-M. (2006). Beyond the Fur paradigm: iron-controlled gene expression in rhizobia. FEMS Microbiol Rev.

[9] Jacquamet L., Aberdam D., Adrait A., Hazemann J.-L., Latour J.-M., Michaud-Soret I. (1998). X-ray absorption spectroscopy of a new zinc site in the fur protein from escherichia coli. Biochemistry.

[10] Pecqueur L., D’Autréaux B., Dupuy J., Nicolet Y., Jacquamet L., Brutscher B., Bersch B. (2006). Structural changes of Escherichia coli ferric uptake regulator during metal-dependent dimerization and activation explored by NMR and x-ray crystallography. J Biol Chem.

[11] Fontenot C.R., Tasnim H., Valdes K.A., Popescu C.V., Ding H. (2020). Ferric uptake regulator (Fur) reversibly binds a [2Fe-2S] cluster to sense intracellular iron homeostasis in Escherichia coli. J Biol Chem.

[12] Fontenot C.R., Ding H. (2023). The C-terminal domain of the ferric uptake regulator (Fur) binds a [2Fe–2S] cluster to sense the intracellular free iron content in Escherichia coli. BioMetals.

[13] Mills S.A., Marletta M.A. (2005). Metal binding characteristics and role of iron oxidation in the ferric uptake regulator from Escherichia coli. Biochemistry.

[14] Deng Z., Wang Q., Liu Z., Zhang M., Machado A.C.D., Chiu T.P., Chen Z. (2015). Mechanistic insights into metal ion activation and operator recognition by the ferric uptake regulator. Nat Commun.

[15] Sheikh M.A., Taylor G.L. (2009). Crystal structure of the Vibrio cholerae ferric uptake regulator (Fur) reveals insights into metal co-ordination. Mol Microbiol.

[16] Lee J.-W., Helmann J.D. (2006). The PerR transcription factor senses H2O2 by metal-catalysed histidine oxidation. Nature.

[17] Bellini P., Hemmings A.M. (2006). In vitro characterization of a bacterial manganese uptake regulator of the fur superfamily. Biochemistry.

[18] Massé E., Gottesman S. (2002). A small RNA regulates the expression of genes involved in iron metabolism in Escherichia coli. Proc Natl Acad Sci USA.

[19] Dubrac S., Touati D. (2000). Fur positive regulation of iron superoxide dismutase in Escherichia coli: Functional analysis of the sodB promoter. J Bacteriol.

[20] Fàbrega A., Vila J. (2013). Salmonella enterica serovar Typhimurium skills to succeed in the host: Virulence and regulation. Clin Microbiol Rev.

[21] Vasil M.L., Ochsner U. a (1999). The response of Pseudomonas aeruginosa to iron: genetics, biochemistry and virulence. Mol Microbiol.

[22] Lucarelli D., Vasil M.L., Meyer-Klaucke W., Pohl E. (2008, August). The metal-dependent regulators FurA and FurB from mycobacterium tuberculosis. Int J Mol Sci.

[23] Wyckoff E.E., Mey A.R., Leimbach A., Fisher C.F., Payne S.M. (2006). Characterization of ferric and ferrous iron transport systems in Vibrio cholerae. J Bacteriol.

[24] van Vliet A.H.M., Stoof J., Vlasblom R., Wainwright S. a, Hughes N.J., Kelly D.J., Kusters J.G. (2002). The role of the Ferric Uptake Regulator (Fur) in regulation of Helicobacter pylori iron uptake. Helicobacter.

[25] Carpenter B.M., Whitmire J.M., Merrell D.S. (2009). This is not your mother’s repressor: The complex role of fur in pathogenesis. Infect Immun.

[26] Hood M.I., Skaar E.P. (2012). Nutritional immunity: transition metals at the pathogen-host interface. Nat Rev Microbiol.

[27] Troxell B., Hassan H.M. (2013). Transcriptional regulation by ferric uptake regulator (Fur) in pathogenic bacteria. Front Cell Infect Microbiol.

[28] Perálvarez-Marín A., Baranowski E., Bierge P., Pich O.Q., Lebrette H. (2021). Metal utilization in genome-reduced bacteria: do human mycoplasmas rely on iron?. Comput Struct Biotechnol J.

[29] Williams K.P., Kelly D.P. (2013). Proposal for a new class within the phylum Proteobacteria, Acidithiobacillia classis nov., with the type order Acidithiobacillales, and emended description of the class Gammaproteobacteria. Int J Syst Evolut Microbiol.

[30] Quatrini R., Johnson D.B. (2019). Acidithiobacillus ferrooxidans. Trends Microbiol.

[31] Yan L., Zhang S., Chen P., Liu H., Yin H., Li H. (2012). Magnetotactic bacteria, magnetosomes and their application. Microbiol Res.

[32] Yang M., Zhan Y., Zhang S., Wang W., Yan L. (2020).

[33] Ingledew W.J. (1982). Thiobacillus Ferrooxidans the bioenergetics of an acidophilic chemolithotroph. BBA Rev Bioenerg.

[34] Andrews S.C., Robinson A.K., Rodríguez-Quiñones F. (2003). Bacterial iron homeostasis. FEMS Microbiol Rev.

[35] Quatrini R., Jedlicki E., Holmes D.S. (2005). Genomic insights into the iron uptake mechanisms of the biomining microorganism Acidithiobacillus ferrooxidans. J Ind Microbiol Biotechnol.

[36] Chen X. ke, Li X. yan, Ha Y. fan, Lin J. qiang, Liu X. mei, Pang X., Chen L. xu (2020). Ferric uptake regulator provides a new strategy for acidophile adaptation to acidic ecosystems. Appl Environ Microbiol.

[37] Sepúlveda-Rebolledo P., González-Rosales C., Dopson M., Pérez-Rueda E., Holmes D.S., Valdés J.H. (2024). Comparative genomics sheds light on transcription factor-mediated regulation in the extreme acidophilic Acidithiobacillia representatives. Res Microbiol.

[38] Quatrini R., Lefimil C., Holmes D.S., Jedlicki E. (2005). The ferric iron uptake regulator (Fur) from the extreme acidophile Acidithiobacillus ferrooxidans. Microbiology.

[39] Ferraz L.F.C., Verde L.C.L., Vicentini R., Felício A.P., Ribeiro M.L., Alexandrino F., Ottoboni L.M.M. (2010). Ferric iron uptake genes are differentially expressed in the presence of copper sulfides in Acidithiobacillus ferrooxidans strain LR. Antonie Van Leeuwenhoek.

[40] Quatrini R., Lefimil C., Veloso F.A., Pedroso I., Holmes D.S., Jedlicki E. (2007). Bioinformatic prediction and experimental verification of Fur-regulated genes in the extreme acidophile Acidithiobacillus ferrooxidans. Nucleic Acids Res.

[41] Li Q., Ren Y., Qiu G., Li N., Liu H., Dai Z., Liu X. (2011). Insights into the pH up-shift responsive mechanism of Acidithiobacillus ferrooxidans by microarray transcriptome profiling. Folia Microbiol.

[42] Dian C., Vitale S., Leonard G. a, Bahlawane C., Fauquant C., Leduc D., Terradot L. (2011). The structure of the Helicobacter pylori ferric uptake regulator Fur reveals three functional metal binding sites. Mol Microbiol.

[43] Sambrook, J., and Russell, D.W. (2001). Molecular Cloning: A Laboratory Manual (3rd ed.). Cold Spring Harbor, NY: Cold Spring Harbor Laboratory Press.

[44] Laemmli U.K. (1970). Cleavage of Structural Proteins during the Assembly of the Head of Bacteriophage T4. Nature.

[45] Bradford M.M. (1976). A rapid and sensitive method for the quantitation of microgram quantities of protein utilizing the principle of protein-dye binding. Anal Biochem.

[46] Wiedemann C., Bellstedt P., Görlach M. (2013). CAPITO - A web server-based analysis and plotting tool for circular dichroism data. Bioinformatics.

[47] Le S.Q., Gascuel O. (2008). An improved general amino acid replacement matrix. Mol Biol Evol.

[48] Tamura K., Stecher G., Kumar S. (2021). MEGA11: Molecular evolutionary genetics analysis version 11. Mol Biol Evol.

[49] Sievers F., Wilm A., Dineen D., Gibson T.J., Karplus K., Li W., Higgins D.G. (2011). Fast, scalable generation of high-quality protein multiple sequence alignments using Clustal Omega. Mol Syst Biol.

[50] Waterhouse A.M., Procter J.B., Martin D.M.A., Clamp M., Barton G.J. (2009). Jalview Version 2-A multiple sequence alignment editor and analysis workbench. Bioinformatics.

[51] Robert X., Gouet P. (2014). Deciphering key features in protein structures with the new ENDscript server. Nucleic Acids Res.

[52] O’Leary N.A., Wright M.W., Brister J.R., Ciufo S., Haddad D., McVeigh R., Pruitt K.D. (2016). Reference sequence (RefSeq) database at NCBI: Current status, taxonomic expansion, and functional annotation. Nucleic Acids Res.

[53] Altschul S.F., Madden T.L., Schäffer A.A., Zhang J., Zhang Z., Miller W., Lipman D.J. (1997). Gapped BLAST and PSI-BLAST: A new generation of protein database search programs. Nucleic Acids Res.

[54] Sali A., Potterton L., Yuan F., van Vlijmen H., Karplus M. (1995). Evaluation of comparative protein modeling by MODELLER. Proteins.

[55] Jumper J., Evans R., Pritzel A., Green T., Figurnov M., Ronneberger O., Hassabis D. (2021). Highly accurate protein structure prediction with AlphaFold. Nature.

[56] Ramachandran G.N., Ramakrishnan C., Sasisekharan V. (1963). Stereochemistry of polypeptide chain configurations. J Mol Biol.

[57] Eisenberg D., Lüthy R., Bowie J.U. (1997). VERIFY3D: assessment of protein models with three-dimensional profiles. Methods Enzymol.

[58] Humphrey W., Dalke A., Schulten K. (1996). VMD: visual molecular dynamics. J Mol Graph.

[59] Phillips J.C., Braun R., Wang W., Gumbart J., Tajkhorshid E., Villa E., Schulten K. (2005). Scalable molecular dynamics with NAMD. J Comput Chem.

[60] Zhu X., Lopes P.E.M., Mackerell A.D. (2012). Recent Developments and Applications of the CHARMM force fields. Wiley Interdiscip Rev Comput Mol Sci.

[61] Morris G.M., Ruth H., Lindstrom W., Sanner M.F., Belew R.K., Goodsell D.S., Olson A.J. (2009). Software news and updates AutoDock4 and AutoDockTools4: Automated docking with selective receptor flexibility. J Comput Chem.

[62] Berman H.M., Westbrook J., Feng Z., Gilliland G., Bhat T.N., Weissig H., Bourne Philip E. (2000). The Protein Data Bank. Nucleic Acids Res.

[63] Khoroshilova N., Popescu C., Münck E., Beinert H., Kiley P.J. (1997). Iron-sulfur cluster disassembly in the FNR protein of Escherichia coli by O 2: [4Fe-4S] to [2Fe-2S] conversion with loss of biological activity. Proc Natl Acad Sci.

[64] Smith D.M.A., Xiong Y., Straatsma T.P., Rosso K.M., Squier T.C. (2012). Force-Field Development and Molecular Dynamics of [NiFe] Hydrogenase. J Chem Theory Comput.

[65] de Hatten X., Cournia Z., Huc I., Smith J.C., Metzler-Nolte N. (2007). Force-field development and molecular dynamics simulations of ferrocene–peptide conjugates as a scaffold for hydrogenase mimics. Chem - A Eur J.

[66] Mitra D., Pelmenschikov V., Guo Y., Case D.A., Wang H., Dong W., Cramer S.P. (2011). Dynamics of the [4Fe-4S] Cluster in Pyrococcus furiosus D14C ferredoxin via nuclear resonance vibrational and resonance raman spectroscopies, force field simulations, and density functional theory calculations. Biochemistry.

[67] Pohl E., Haller J.C., Mijovilovich A., Meyer-Klaucke W., Garman E., Vasil M.L. (2003). Architecture of a protein central to iron homeostasis: crystal structure and spectroscopic analysis of the ferric uptake regulator. Mol Microbiol.

[68] D’Autréaux B., Pecqueur L., De Peredo A.G., Diederix R.E.M., Caux-Thang C., Tabet L., Michaud-Soret I. (2007). Reversible redox- and zinc-dependent dimerization of the Escherichia coli fur protein. Biochemistry.

[69] Ahmad R., Brandsdal B.O., Michaud-Soret I., Willassen N.P. (2009). Ferric uptake regulator protein: Binding free energy calculations and per-residue free energy decomposition. Protein: Struct, Funct Bioinforma.

[70] Arenas-Salinas M., Ortega-Salazar S., Gonzales-Nilo F., Pohl E., Holmes D.S., Quatrini R. (2014). AFAL: a web service for profiling amino acids surrounding ligands in proteins. J Comput-Aided Mol Des.

[71] Angles R., Arenas-Salinas M., García R., Reyes-Suarez J.A., Pohl E. (2020). GSP4PDB: a web tool to visualize, search and explore protein-ligand structural patterns. BMC Bioinforma.

[72] Beinert H., Emptage M.H., Dreyer J.L., Scott R. a, Hahn J.E., Hodgson K.O., Thomson a J. (1983). Iron-sulfur stoichiometry and structure of iron-sulfur clusters in three-iron proteins: evidence for [3Fe-4S] clusters. Proc Natl Acad Sci USA.

[73] Bingemann R., Klein A. (2000). Conversion of the central [4Fe-4S] cluster into a [3Fe-4S] cluster leads to reduced hydrogen-uptake activity of the F420-reducing hydrogenase of Methanococcus voltae. Eur J Biochem.

[74] Zeng J., Liu Q., Zhang X., Mo H., Wang Y., Chen Q., Liu Y. (2010). Functional roles of the aromatic residues in the stabilization of the [Fe4S4] cluster in the Iro protein from Acidithiobacillus ferrooxidans. J Microbiol Biotechnol.

[75] Liu Y., from A. ferrooxidans O. T. I. B. M. B. B. O. O. V. C. B. [4Fe–4S] and [3Fe–4S], Guo S., Yu R., Ji J., Qiu G. (2013). HdrC2 from acidithiobacillus ferrooxidans owns two iron–sulfur binding motifs but binds only one variable cluster between [4Fe–4S] and [3Fe–4S]. Curr Microbiol.

[76] Dai Y., Liu J., Zheng C., Wu A., Zeng J., Qiu G. (2009). Cys92, Cys101, Cys197, and Cys203 Are crucial residues for coordinating the iron–sulfur cluster of rhda from acidithiobacillus ferrooxidans. Curr Microbiol.

[77] Zeng J., Jiang H., Liu Y., Liu J., Qiu G. (2008). Expression, purification and characterization of a high potential iron-sulfur protein from Acidithiobacillus ferrooxidans. Biotechnol Lett.

[78] Nishio K., Nakai M. (2000). Transfer of iron-sulfur cluster from NifU to apoferredoxin. J Biol Chem.

[79] Jin Z., Heinnickel M., Krebs C., Shen G., Golbeck J.H., Bryant D.A. (2008). Biogenesis of iron-sulfur clusters in photosystem I: Holo-NfuA from the cyanobacterium Synechococcus sp. PCC 7002 rapidly and efficiently transfers [4Fe-4S] clusters to apo-PsaC in vitro. J Biol Chem.

[80] Strange R.W., Blackburn N.J., Knowles P.F., Hasnain S.S. (1987). X-ray Absorption spectroscopy of metal-histidine coordination in metalloproteins. exact simulation of the EXAFS of Tetrakis(imidazole)copper(II) nitrate and other copper-imidazole complexes by the use of a multiple-scattering treatment. J Am Chem Soc.

[81] Lucarelli D., Russo S., Garman E., Milano A., Meyer-Klaucke W., Pohl E. (2007). Crystal structure and function of the zinc uptake regulator FurB from Mycobacterium tuberculosis. J Biol Chem.

[82] Newville M. (2001). IFEFFIT: Interactive XAFS analysis and FEFF Fitting. *J Synchrotron Rad*.

[83] Fontenot C.R., Ding H. (2022). Ferric uptake regulators (Fur) from Vibrio cholerae and Helicobacter pylori bind a [2Fe–2S] cluster in response to elevation of intracellular free iron content. BioMetals.

[84] Pérard J., Covès J., Castellan M., Solard C., Savard M., Miras R., De Rosny E. (2016). Quaternary structure of fur proteins, a new subfamily of tetrameric proteins. Biochemistry.

[85] Gilston B.A., Wang S., Marcus M.D., Canalizo-Hernández M.A., Swindell E.P., Xue Y., O’Halloran T.V. (2014). Structural and mechanistic basis of zinc regulation across the E. coli Zur regulon. PLoS Biol.

[86] Shin J.-H., Jung H.J., An Y.J., Cho Y.-B., Cha S.-S., Roe J.-H. (2011). Graded expression of zinc-responsive genes through two regulatory zinc-binding sites in Zur. Proc Natl Acad Sci USA.

[87] Makthal N., Rastegari S., Sanson M., Ma Z., Olsen R.J., Helmann J.D., Kumaraswami M. (2013). Crystal structure of peroxide stress regulator from streptococcus pyogenes provides functional insights into the mechanism of oxidative stress sensing. J Biol Chem.

[88] Sarvan S., Charih F., Butcher J., Brunzelle J.S., Stintzi A., Couture J.F. (2018). Crystal structure of campylobacter jejuni peroxide regulator. FEBS Lett.

[89] Pérard J., Nader S., Levert M., Arnaud L., Carpentier P., Siebert C., Michaud-Soret I. (2018). Structural and functional studies of the metalloregulator Fur identify a promoter-binding mechanism and its role in Francisella tularensis virulence. Commun Biol.

[90] Beinert H. (2000). Iron-sulfur proteins: ancient structures, still full of surprises. J Biol Inorg Chem.

[91] Müllner M., Hammel O., Mienert B., Schlag S., Bill E., Unden G. (2008). A PAS domain with an oxygen labile [4Fe-4S]2+ cluster in the oxygen sensor kinase NreB of staphylococcus carnosus. Biochemistry.

[92] Gruner I., Frädrich C., Böttger L.H., Trautwein A.X., Jahn D., Härtig E. (2011). Aspartate 141 is the fourth ligand of the oxygen-sensing [4Fe-4S] 2+ cluster of Bacillus subtilis transcriptional regulator Fnr. J Biol Chem.

[93] Bush M., Ghosh T., Tucker N., Zhang X., Dixon R. (2011). Transcriptional regulation by the dedicated nitric oxide sensor, NorR: a route towards NO detoxification. Biochem Soc Trans.

[94] Pinochet-Barros A., Helmann J.D. (2018). Redox sensing by Fe2+ in bacterial fur family metalloregulators. Antioxid Redox Signal.

[95] Fernández-Otal Á., Guío J., Sarasa-Buisan C., Peleato M.L., Fillat M.F., Lanas Á., Bes M.T. (2024). Functional characterization of Fur from the strict anaerobe Clostridioides difficile provides insight into its redox-driven regulatory capacity. FEBS J.

[96] Kandari D., Joshi H. (2024). PerR: A peroxide sensor eliciting metal ion-dependent regulation in various bacteria. Mol Biotechnol.

[97] Butcher J., Sarvan S., Brunzelle J.S., Couture J.-F., Stintzi A. (2012). Structure and regulon of Campylobacter jejuni ferric uptake regulator Fur define apo-Fur regulation. Proceedings of the National Academy of Sciences of the United States of America.

