# Supplementary material for: Novel metal sites revealed by spectroscopic and structural characterization of the ferric uptake regulator from Acidithiobacillus ferrooxidans
Source: Comput Struct Biotechnol J. 2025 Feb 19;27:765–77. doi: 10.1016/j.csbj.2025.02.017 (PMC11910503; doi:10.1016/j.csbj.2025.02.017)
Supplement: table S1 — Supplementary material [file mmc1.docx]

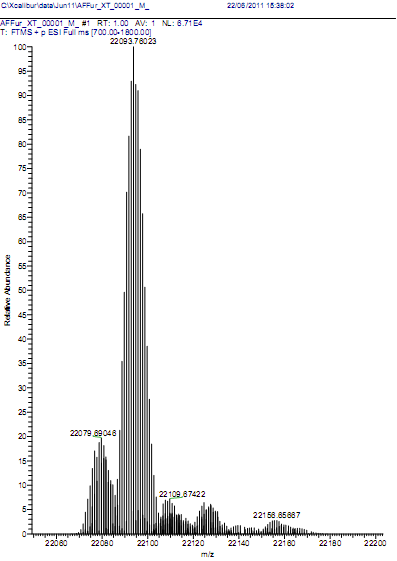


**Figure S1. The molecular weight and purity of the purified protein was determined by mass spectrometry**, after the second chromatographic step. The main component of this fraction has a molecular weight of 22093.76 Da. This value is close to the estimated theoretical size for the recombinant AfFur protein (22063.7 Da) considering the difference in size originating from the additional amino acids incorporated from the poly-histidine domain at the N-terminus from pLate51 vector. This component is 90% pure.


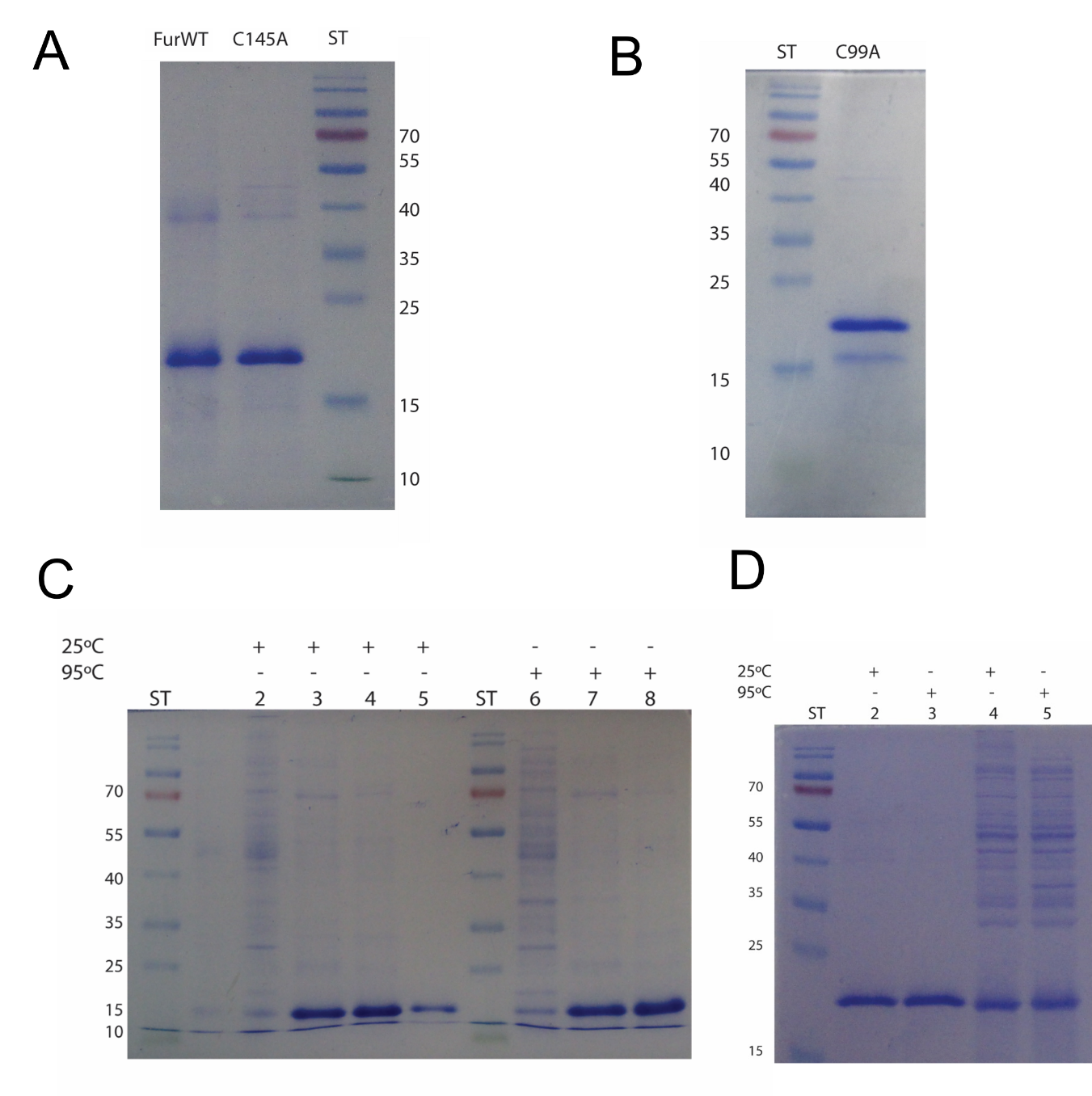


**Figure S2: SDS-PAGE 16% gel electrophoresis of recombinant wild type AfFur and AfFur mutant proteins**. (A and B) Purified protein preparations checked electrophoretically and stained with Coomasie blue. (C and D) Purification using HisTrapTMHP affinity chromatography were. (**A**) Lanes: AfFur (FurWT), mutant C145A. (**B**) Mutant C99A. (**C**) Lanes 2 and 6: C136A SN lysate. Lanes 3 and 7: purified C136A. Lanes 4 and 8: FurWT. Lane 5: C99A. (**D**) Purification of mutant Fur protein C99A through affinity chromatography. HisTrap^TM^HP A) SDS-PAGE 16%. 2 μg of protein were loaded in each lane. Abbreviations: ST: molecular weight marker, kDa.


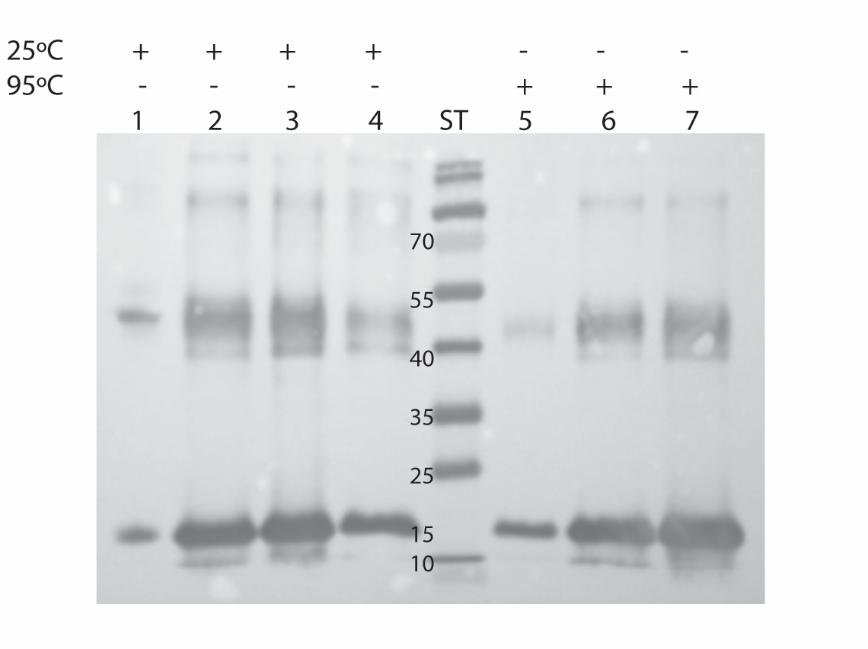

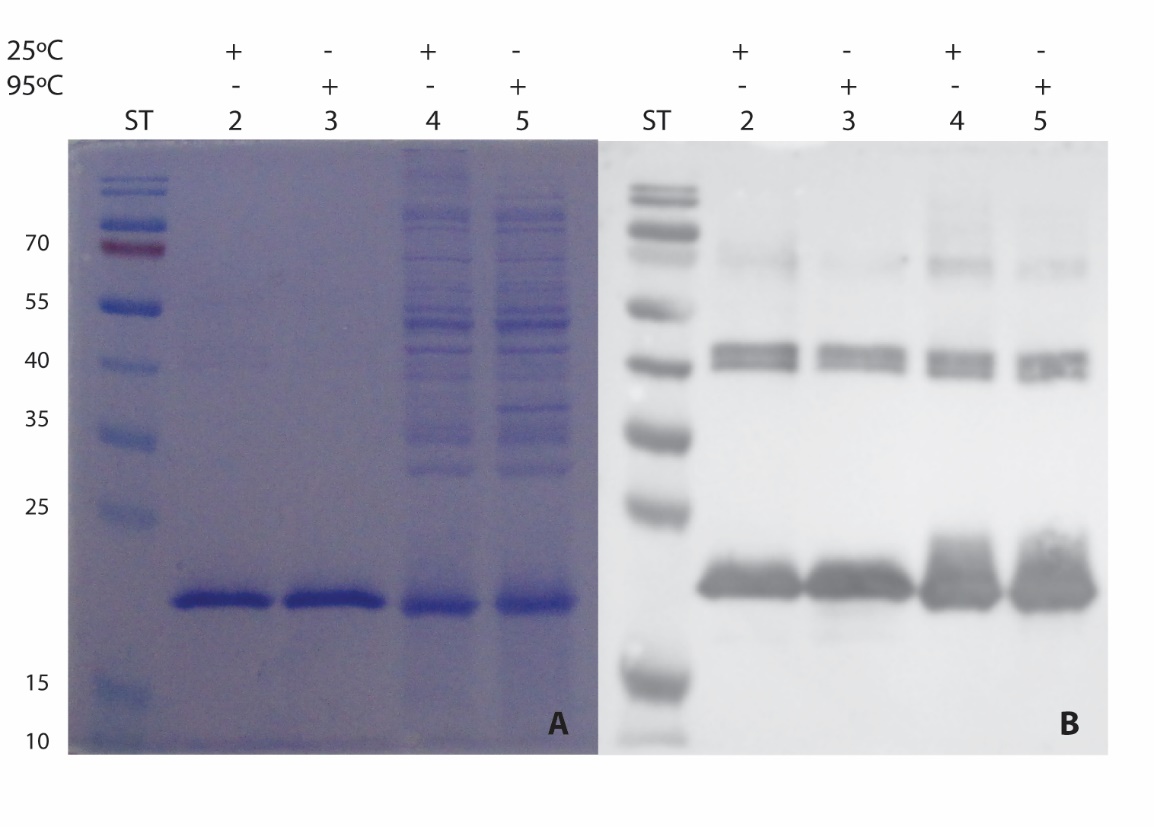


A

B

**Figure S3: Western blot of recombinant wild type and mutant AfFur protein preparations**. A: Lanes 1 and 5: C136A SN lysate. Lanes 2 and 6: purified C136A. Lanes 3 and 7: FurWT. Lane 4: C99A. 2 μg of protein by lane. B: western blot. Lanes 2-3: purified C99A, 2.8 μg. Lanes 4-5: lysated C99A. Blots were then incubated with primary monoclonal antibody 6X-His Tag Mab (MA121315 Thermofisher) at 1/2500 in blocking solution for 1 h at RT. Blots were washed 4 times for 5 min in TBS-T at RT, and incubated with secondary antibody AP-conjugated Goat Anti-Mouse IgG at 1/1000 in blocking solution for 1 h at RT. Blots were washed 4 times for 5 min in TBS-T and developed for 5 min at RT with Promega BCIP/NBT Color Development Substrate. Abbreviations: ST, molecular weight marker, kDa.


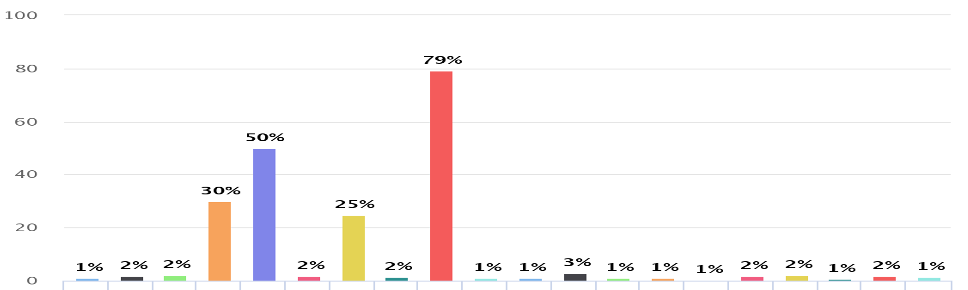

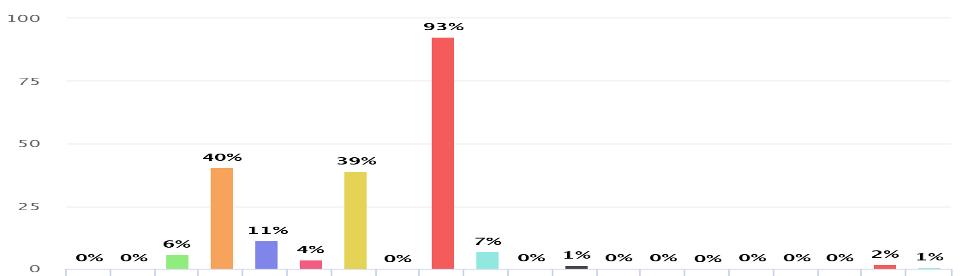

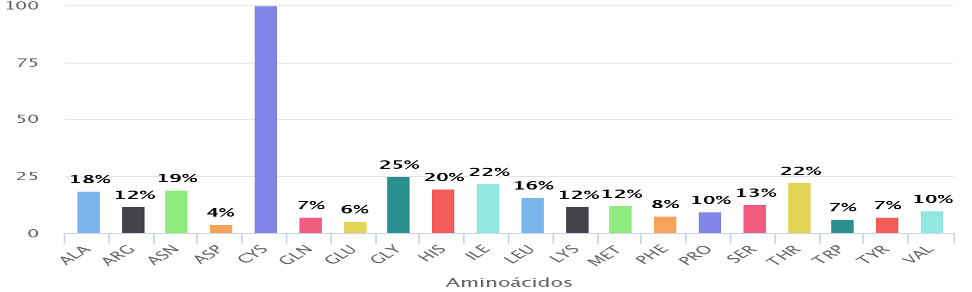


**[4Fe-4S] clusters (PDB code: SF4)**

**Fe^+2^ (PDB code: FE2)**

**Zn^2+^ (PDB code: ZN2)**

**Cys**

**His**

**Glu**

**Asp**

**Ile**

**Leu**

**Lys**

**Met**

**Phe**

**Pro**

**Ser**

**Thr**

**Trp**

**Tyr**

**Val**

**Ala**

**Arg**

**Asn**

**Gln**

**Gly**

Frequency of amino acids in the coordination surrounding (%)

**A**

**B**

**C**

**Figure S4. Frequency of amino acids surrounding the coordination of different candidate AfFur metal coregulators.** Proteins obtained from PDB (may 2023) were analyzed with the AFAL2 software (Arenas-Salinas et al., 2014) and the frequency of each amino acid in a 3 Å selection sphere was calculated. Analysis of crystalized protein containing: **(A)** Zn^+2^ atoms (PDB code: ZN2), (**B**) Fe^+2^ atoms (PDB code: FE2) and (**C**) [4Fe-4S] clusters (PDB code: SF4). Most frequent residues (>30%) are highlighted in color (grey:metal atoms; orange:Fe-S clusters)


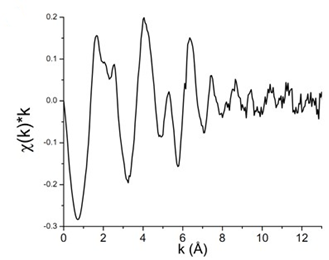


**Figure S5.** **Characterization of the iron-binding microenvironment using EXAFS.** The Extended X-ray Absorption Fine Structure (EXAFS) spectrum for the iron-binding protein is shown. The x-axis (k) represents the wave number in Å⁻¹, which is proportional to the square root of the energy above the iron absorption edge. The y-axis (χ(k) * k) depicts the product of the oscillation function (χ(k)) and the wave number (k), highlighting the EXAFS oscillations that indicate the presence and distances of neighboring atoms around the iron atom. Peaks and valleys in the spectrum correspond to interference patterns caused by electron scattering from atoms surrounding the iron, providing insights into the local atomic environment and coordination geometry.


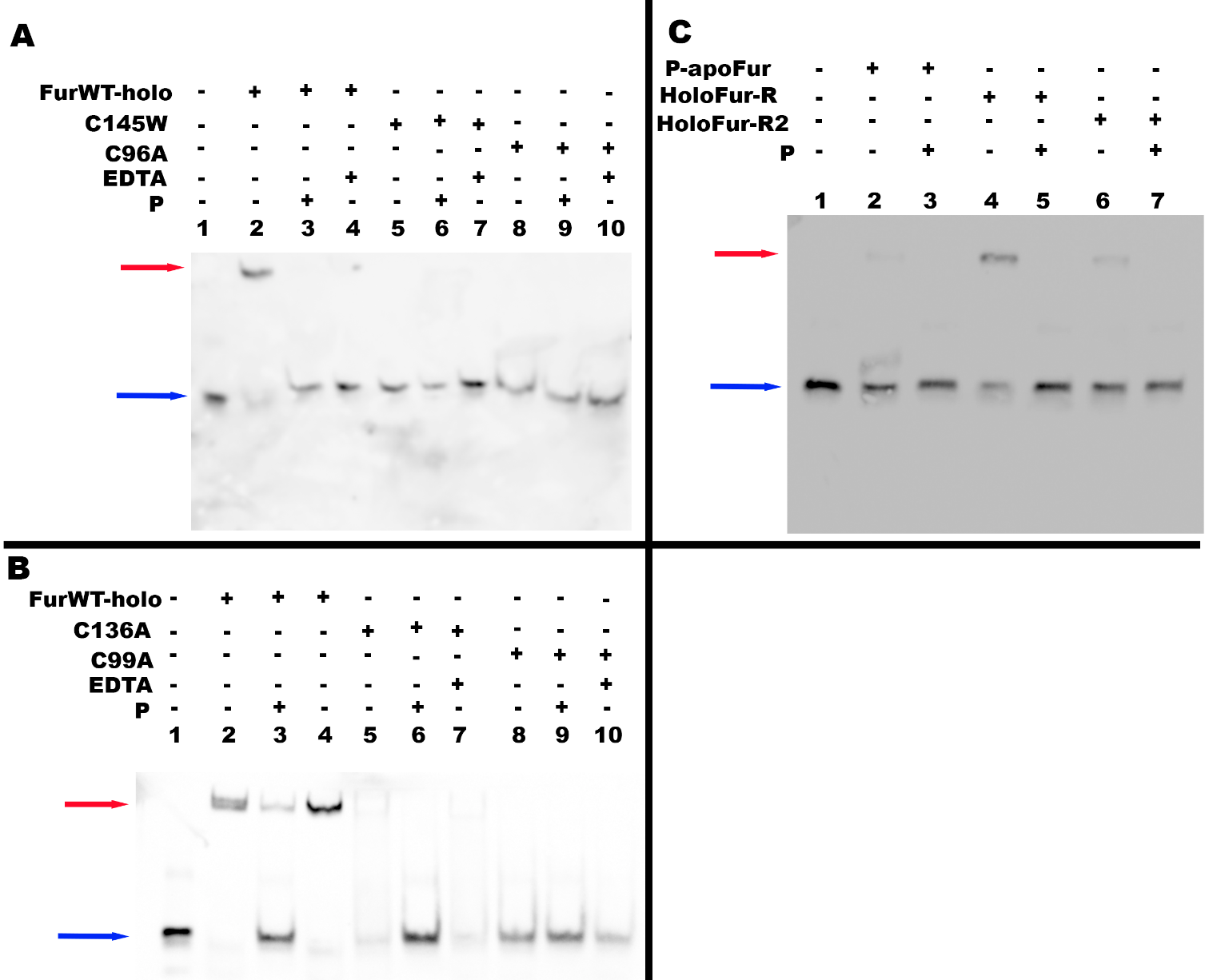


**Figure S6**: **Electrophoretic mobility shift assays (EMSA) of AfFur proteins with a known target Fur box.** (A) Determination of the ability of AfFur wild type (**FurWT-holo**) and mutants **C145W** and **C96A** to bind the Fur box (mntH gene). (B) Mutants C136A and C99A. (C) Binding of AfFur wild type to the Fur box depends on the presence of metal cofactors.

The **partially apo protein**, without the metallic atom (**P-apoFur**) was obtained by incubating AfFur with EDTA and DTT at room temperature. Then the partially apo Fur form was reconstituted by incubating the protein with Fe^2+^ (**HoloFur-R**). For **HoloFur-R2**, the excess of Fe^2+^ was eliminated by the Amicon filter 10kDa. Probe DNA was incubated with 2.9 μg of purified protein. P: competing excess of unlabeled probe of DNA; Plus sign (+): presence of the condition; Minus sign (-): absence of the condition. Red arrow show the shift. Blue arrow show the probe.


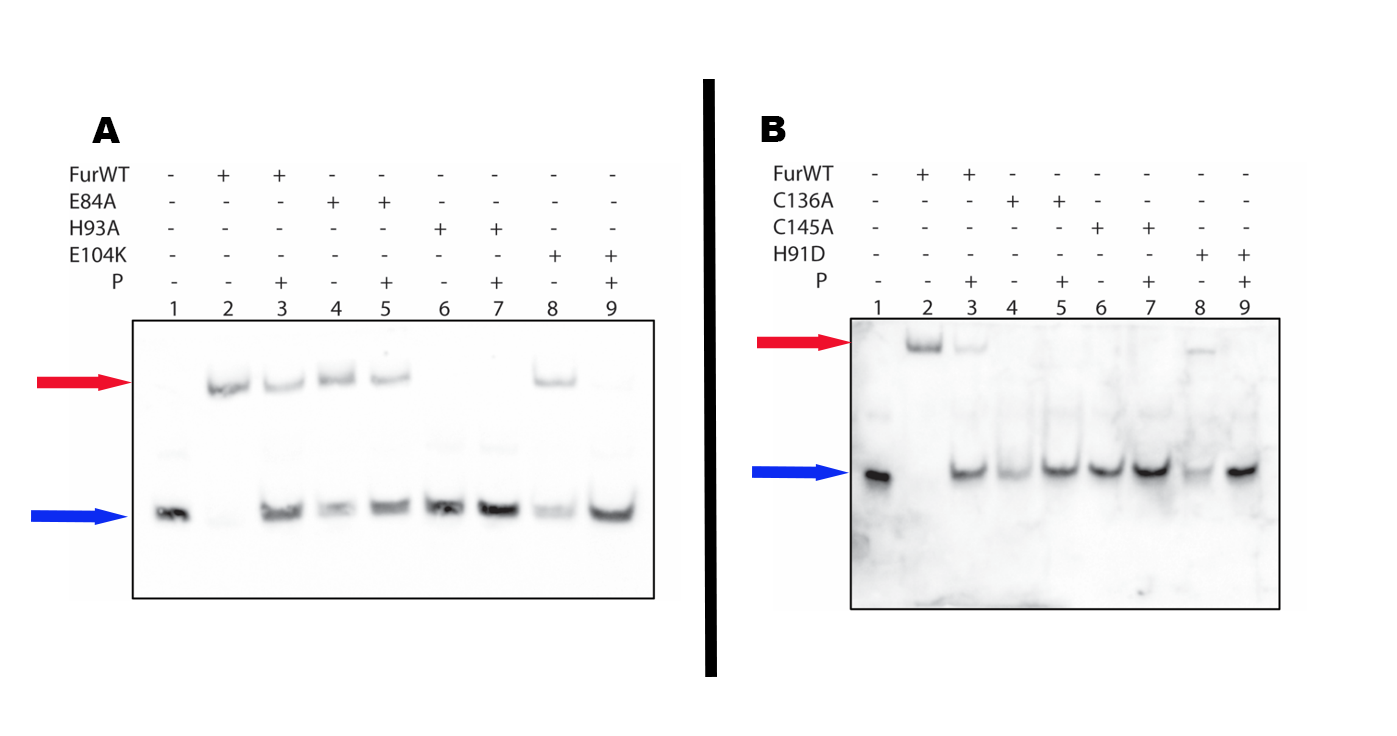


**Figure S7: Electrophoretic mobility shift assays (EMSA) of AfFur proteins with a known target Fur box.** (A) AfFur wild type (**FurWT**) and mutants E84A, H93A, and E104K. (B) FurWT and mutants H91D, C136A and C145A. Probe DNA was incubated with 2.9 μg of purified protein. P: competing excess of unlabeled probe of DNA; Plus sign (+): presence of the condition; Minus sign (-): absence of the condition. Red arrow show the shift. Blue arrow show the probe.

FurWT - + - - + + -

H91K + - + + - - -

EDTA + + - - - - -

P* - - + - + - -

P + + + + + + +

1 2 3 4 5 6 7


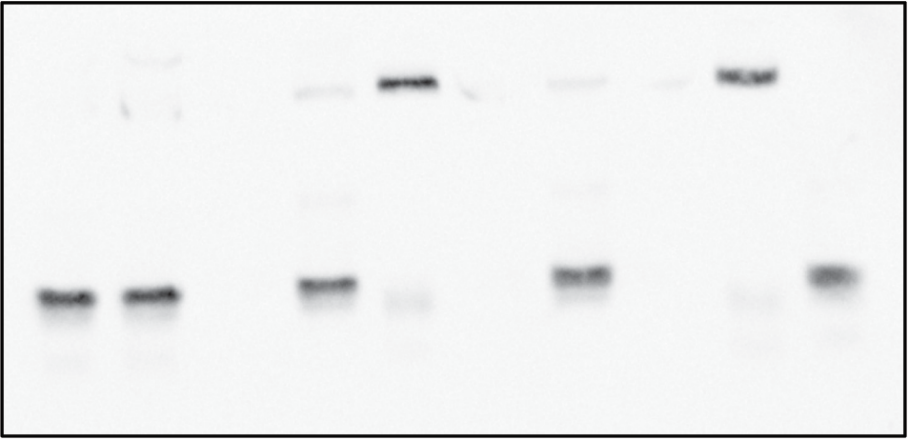


**Figure S8:** **EMSA study of the ability of AfFur wild type (FurWT) and mutant H91K to bind to Fur box**. Probe DNA was incubated with 3.9 and 3.5 μg of purified protein. P: probe DNA, 5’biotin-Furbox; P*: competing excess of unlabelled probe of DNA; Plus sign (+): protein is present; Minus sign (-): protein is not present. EDTA 1mM.


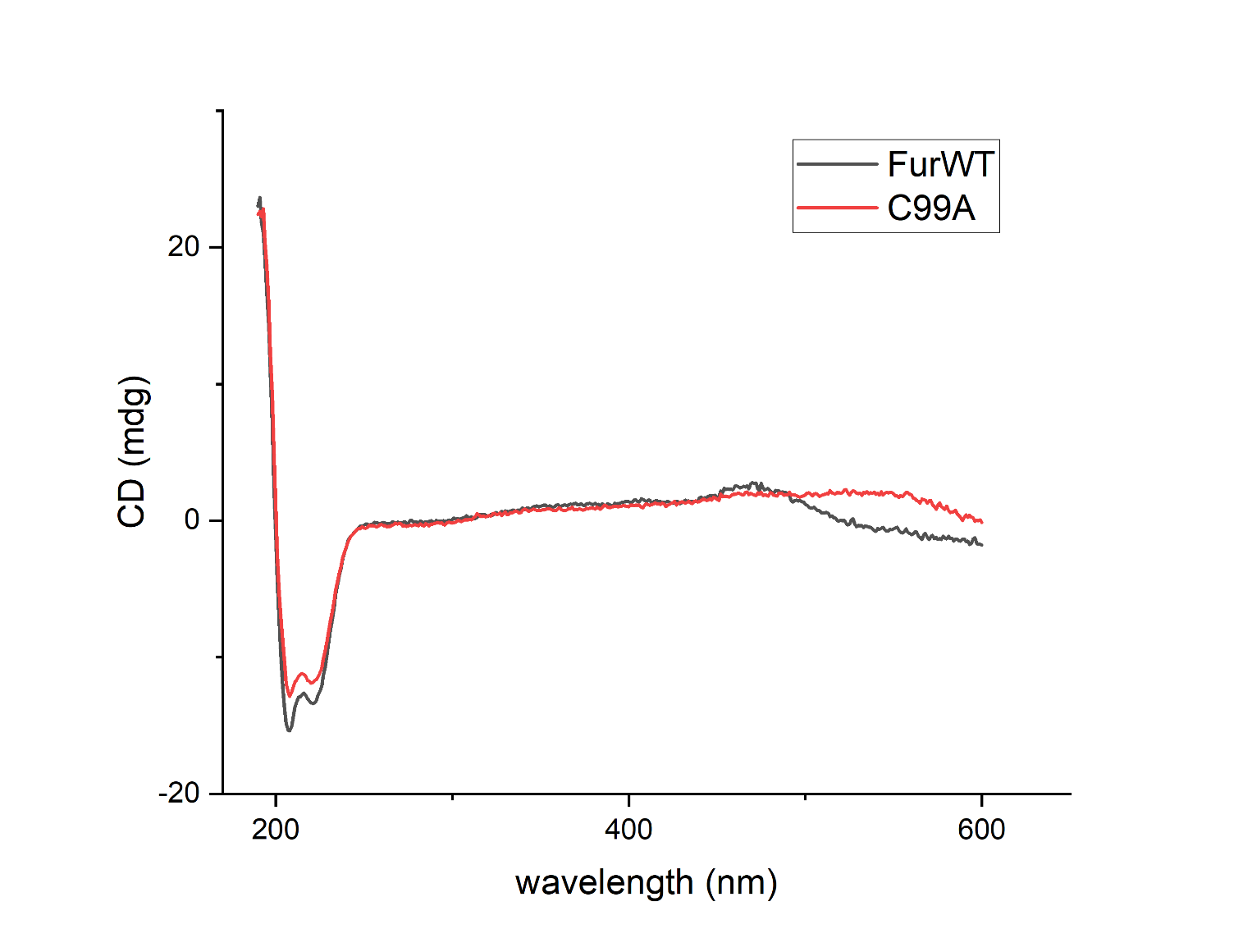


**Figure S9.** The circular dichroism (CD) spectra of AfFur wild type (black) and the C99A mutant (red) are shown. The CD measurements were recorded in the wavelength range of 200-600 nm, indicating no significative changes in the secondary structure between the wild type and mutant proteins.


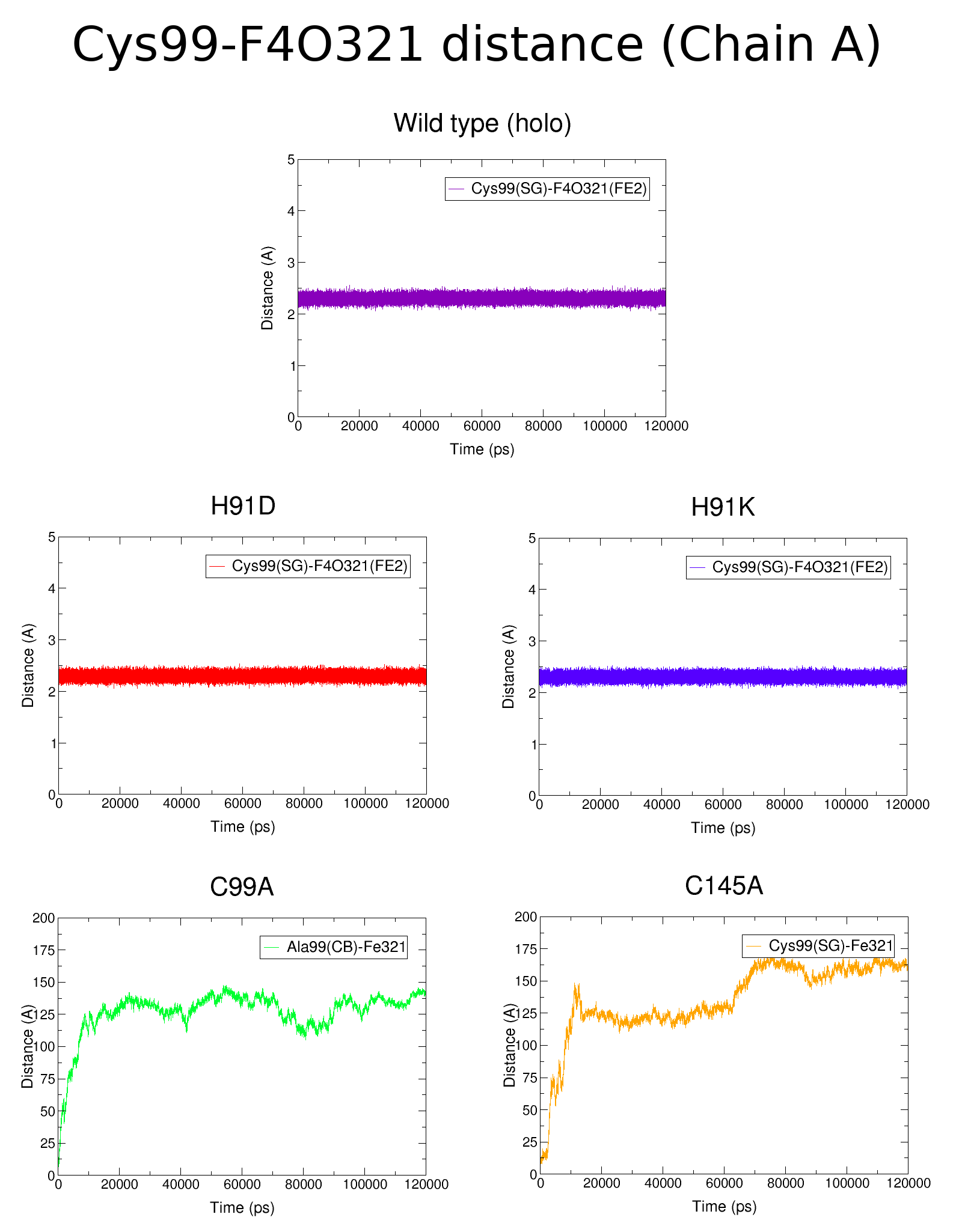


**Figure S10:** Distance (Å) during molecular dynamics between residues C99 (site 3 of chain A) and **4Fe-4S cluster** (id: 321) of wild type AfFur (purple) and mutants H91D (red), H91K (blue), C99A (green) and C145A (orange).


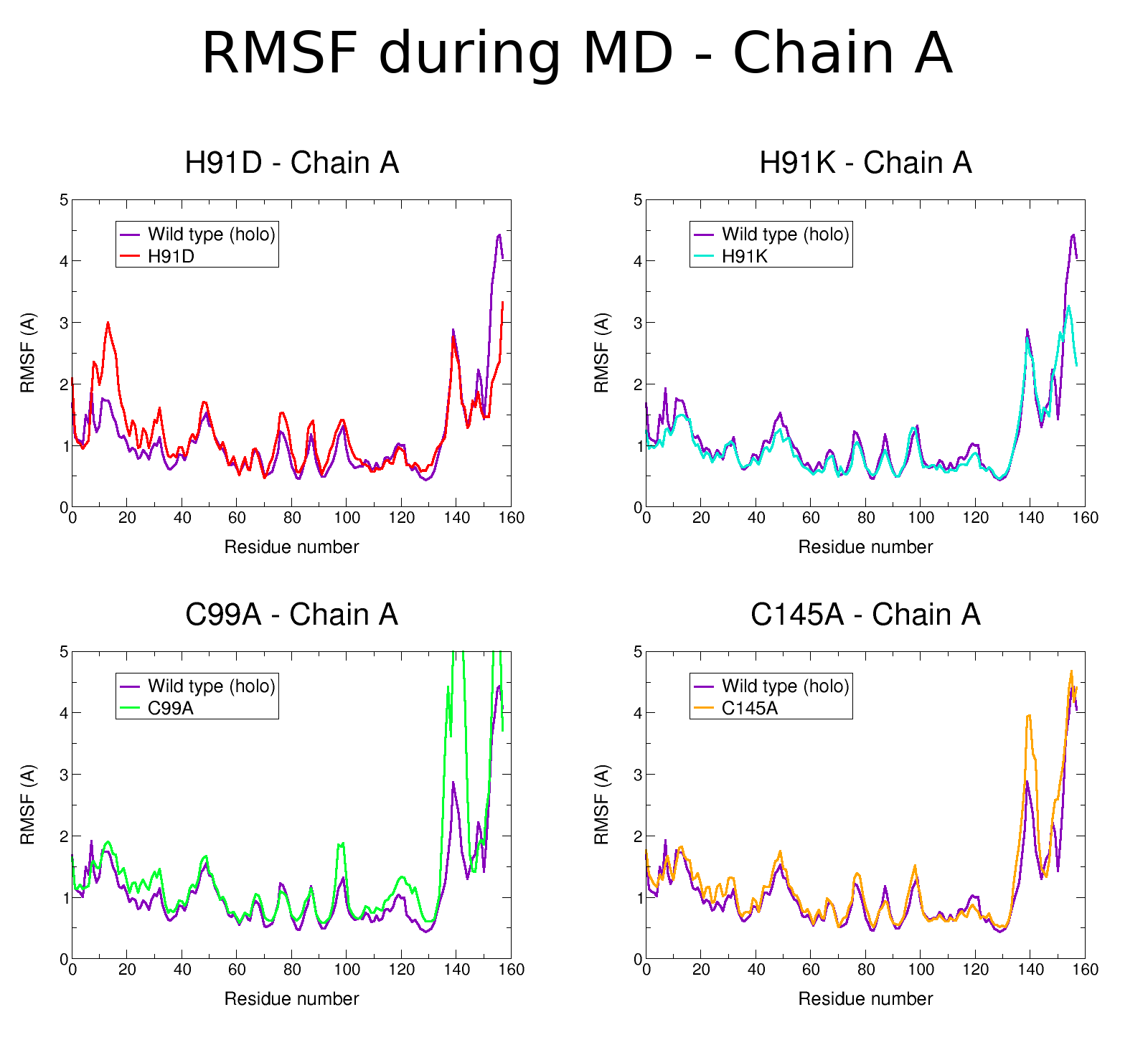
**Figure S11:** Root mean square fluctuation (RMSF) of residues from chain A of AfFur mutants H91D (red), H91K (light blue), C99A (green) and C145A (orange), in comparison to those of residues from chain A of wild type AfFur (purple).


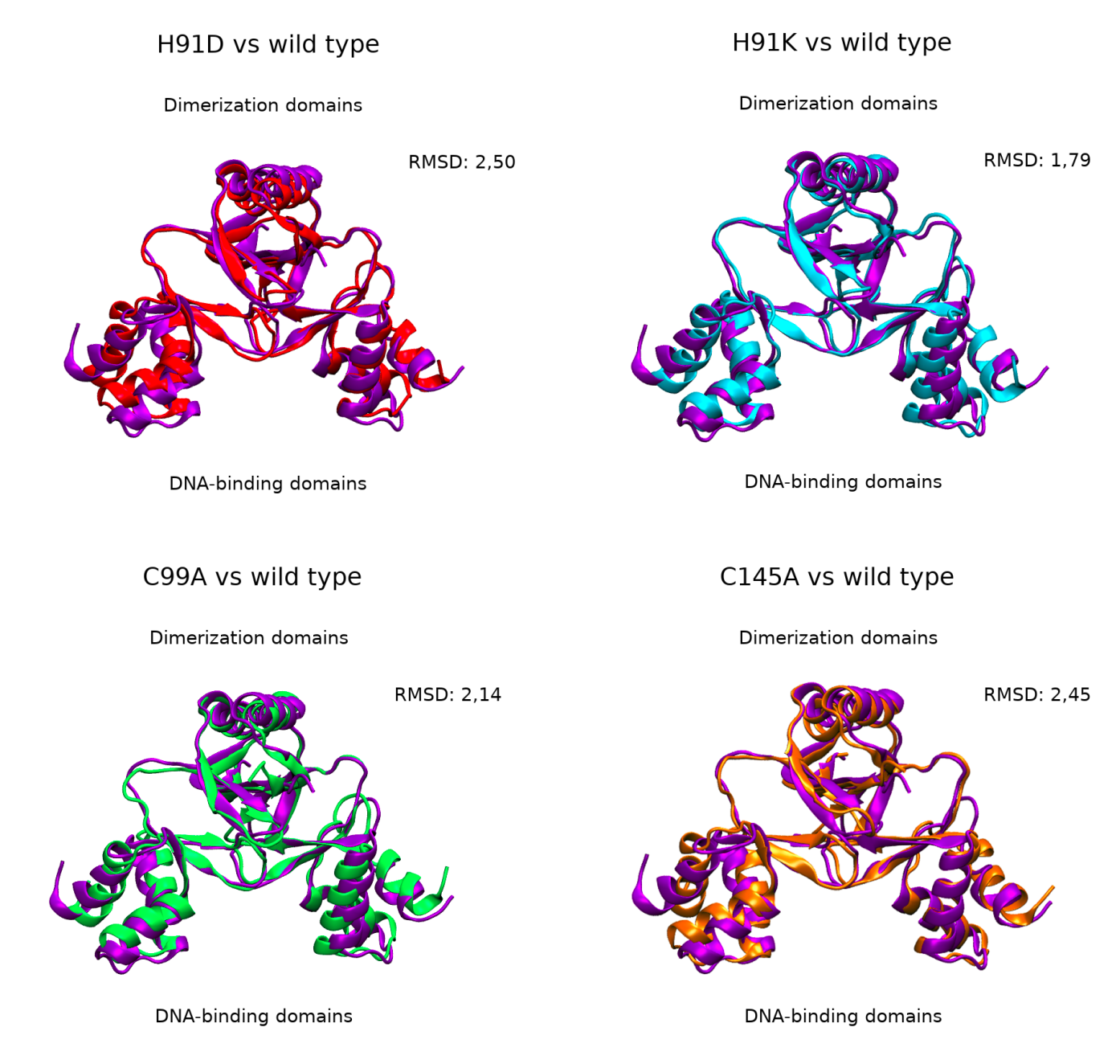


**Figure S12:** Structural superposition of AfFur mutants H91D (red), H91K (blue), C99A (green) and C145A (orange) over wild type AfFur (purple).


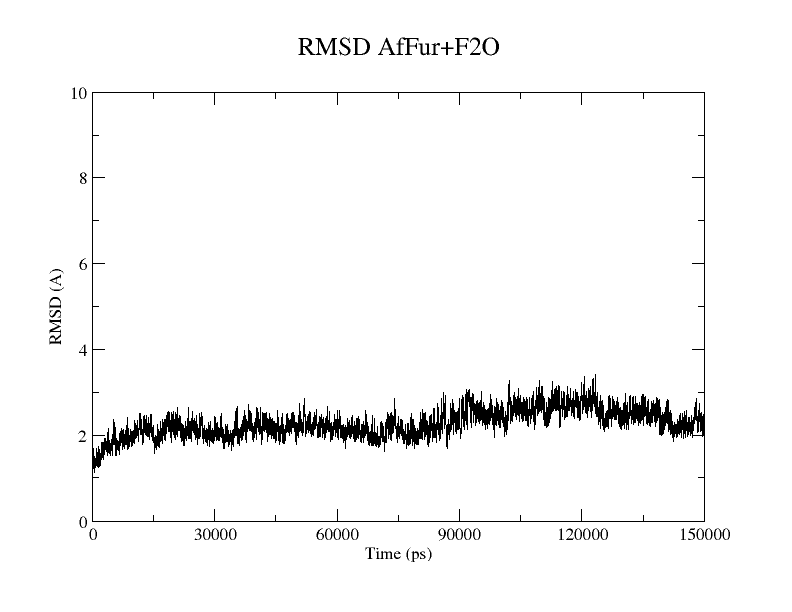


**Figure S13**: Root mean square deviation (RMSD) of AfFur and the [2Fe-2S] cluster (code F2O).


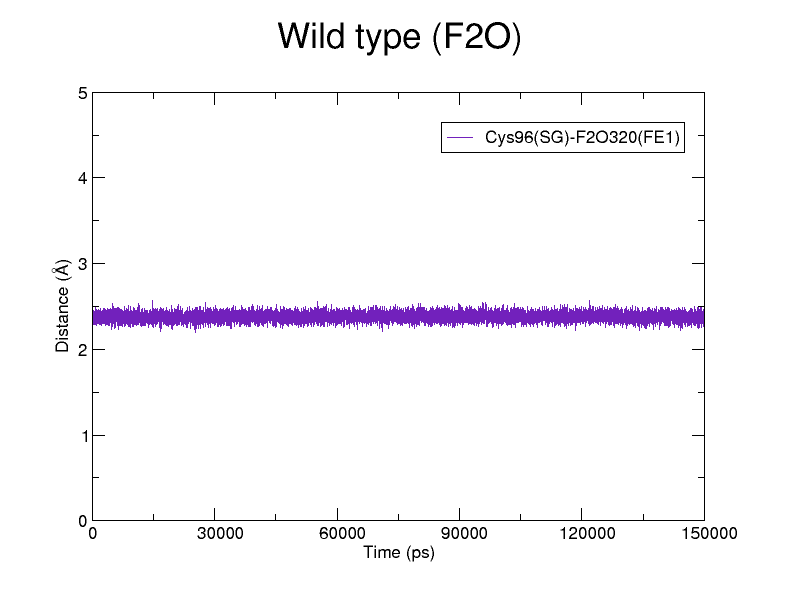

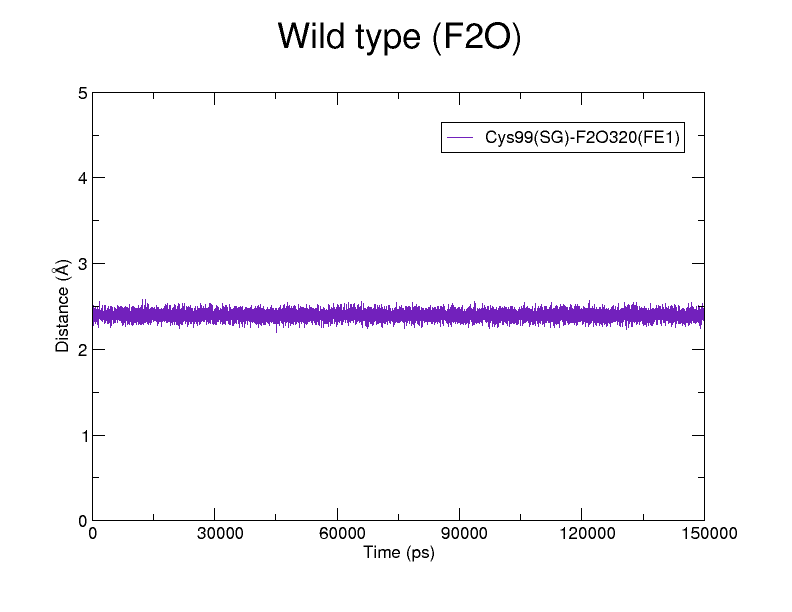


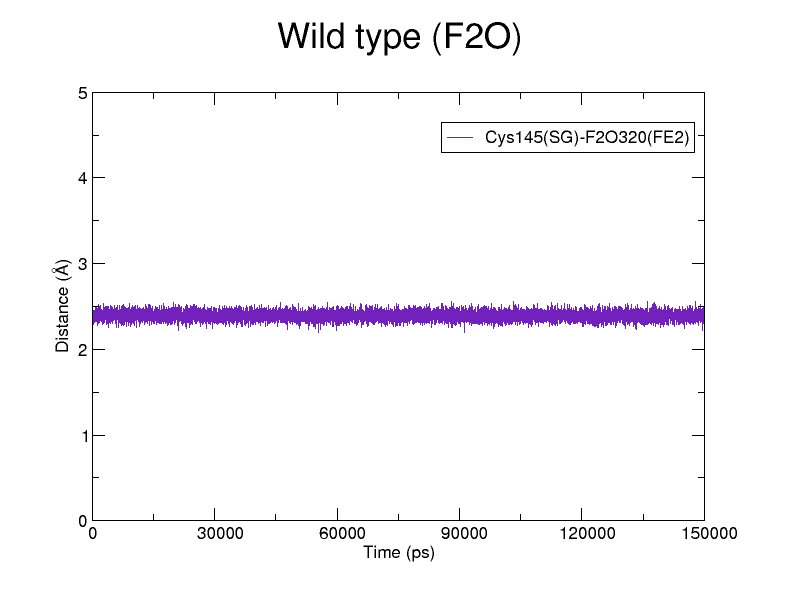

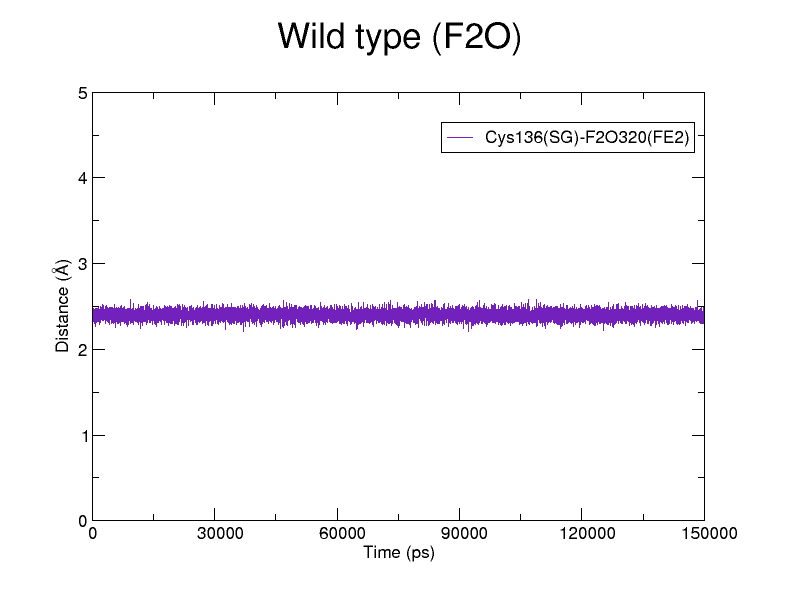


**Figure S14**: Distance (Å) during molecular dynamics between residues C96, C99, C133 and C145 and **[2Fe-2S] cluste**r of wild type AfFur.


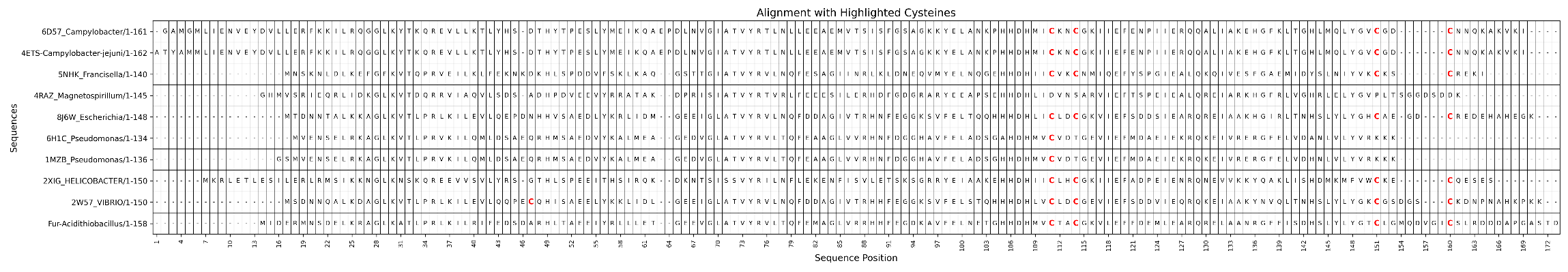


**Figure S15:** Amino acid sequence alignment between AfFur and Fur proteins with known 3D structure. Cysteines are highlighted in red.

**Table S1:** sequence code and organism name

| **Access code** | **Organism Name** |
| --- | --- |
| WP_003242495.1 | *Acidithiobacillus thiooxidans* |
| WP_003242493.1 | *Acidithiobacillus ferrooxidans* |
| WP_010960372.1 | *Acidithiobacillus ferrivorans* |
| WP_012907473.1 | *Acidithiobacillus caldus* |
| WP_016748319.1 | *Sulfuriferula multivorans* |
| WP_045686318.1 | *Sulfuriferula denitrificans* |
| WP_012907472.1 | *Thermithiobacillus tepidarius* |
| WP_010960373.1 | *Thermithiobacillus plumbophilus* |
| WP_046268904.1 | *Halothiobacillus neapolitanus* |
| WP_046268905.1 | *Halothiobacillus kellyi* |
| WP_011288787.1 | *Thioalkalivibrio nitratireducens* |
| WP_011288788.1 | *Thioalkalivibrio thiocyanodenitrificans* |
| WP_013159729.1 | *Thiomonas intermedia* |
| WP_013159728.1 | *Thiomonas delicata* |
| WP_013076247.1 | *Thiobacillus denitrificans* |
| WP_013076246.1 | *Thiobacillus thioparus* |
| WP_016801422.1 | *Starkeya novella* |
| WP_016801421.1 | *Starkeya rubra* |
| WP_011745596.1 | *Xanthobacter autotrophicus* |
| WP_011745595.1 | *Xanthobacter flavus* |

| **AfFur** | **Site 1** |
| --- | --- |
| Wild type | Glu84, His91**, His93**, 2 H_2_O |
| H91D | His36, Glu94, His91, **His93**, Glu104 |
| H91K | His74, Glu84, **His93**, 3 H_2_O |
| C99A | His36, Glu94, His91, **His93**, Glu104 |
| C145A | His36, Glu94, **His93**, Glu104, 1 H_2_O |

**Table S2.** Residues coordinating the iron ion in metal binding site 1 of wild type AfFur and mutants H91D, H91K, C99A and C145A after molecular dynamics.

**Table S3:** Fur superfamily proteins aminoacidic sequence identity matrix. Analysis was performed with Clustal Omega (Chenna et al., 2003)

|  | ScNur | MtZur | ScZur | CjFur | HpFur | MgFur | RlMur | FtFur | AfFur | VcFur | PaFur | LiPerR | CjPerR | BsPerR | SpPerR |
| --- | --- | --- | --- | --- | --- | --- | --- | --- | --- | --- | --- | --- | --- | --- | --- |
| ScNur |  | 31,54 | 24,09 | 28,97 | 21,28 | 20,98 | 23,74 | 26,09 | 23,61 | 27,86 | 26,87 | 17,65 | 24,09 | 25,53 | 21,38 |
| MtZur | 31,54 |  | 58,02 | 28,24 | 25,95 | 26,72 | 31,3 | 25,19 | 32,82 | 35,38 | 31,25 | 19,2 | 20,93 | 27,69 | 25,19 |
| ScZur | 24,09 | 58,02 |  | 28,26 | 21,9 | 21,74 | 27,41 | 21,32 | 28,26 | 31,11 | 27,48 | 14,39 | 21,05 | 24,82 | 25,36 |
| CjFur | 28,97 | 28,24 | 28,26 |  | 38,67 | 37,93 | 34,51 | 30,94 | 31,97 | 34,75 | 36,3 | 22,76 | 26,81 | 29,66 | 20,51 |
| HpFur | 21,28 | 25,95 | 21,9 | 38,67 |  | 30,07 | 27,46 | 32,37 | 23,78 | 29,2 | 27,41 | 15,97 | 23,13 | 22,22 | 21,33 |
| MgFur | 20,98 | 26,72 | 21,74 | 37,93 | 30,07 |  | 60,99 | 30,94 | 31,94 | 38,85 | 39,26 | 22,46 | 19,12 | 27,27 | 16,55 |
| RlMur | 23,74 | 31,3 | 27,41 | 34,51 | 27,46 | 60,99 |  | 30,43 | 34,75 | 36,03 | 36,3 | 20,59 | 20,9 | 26,24 | 19,01 |
| FtFur | 26,09 | 25,19 | 21,32 | 30,94 | 32,37 | 30,94 | 30,43 |  | 35 | 38,69 | 40,74 | 23,31 | 26,32 | 26,09 | 23,02 |
| **AfFur** | **23,61** | **32,82** | **28,26** | **31,97** | **23,78** | **31,94** | **34,75** | **35** |  | **52,03** | **53,68** | **18,84** | **25,36** | **30,07** | **22,73** |
| VcFur | 27,86 | 35,38 | 31,11 | 34,75 | 29,2 | 38,85 | 36,03 | 38,69 | 52,03 |  | 54,81 | 23,48 | 25,74 | 25,55 | 22,92 |
| PaFur | 26,87 | 31,25 | 27,48 | 36,3 | 27,41 | 39,26 | 36,3 | 40,74 | 53,68 | 54,81 |  | 22,48 | 23,08 | 24,63 | 21,48 |
| LiPerR | 17,65 | 19,2 | 14,39 | 22,76 | 15,97 | 22,46 | 20,59 | 23,31 | 18,84 | 23,48 | 22,48 |  | 23,48 | 28,78 | 23,45 |
| CjPerR | 24,09 | 20,93 | 21,05 | 26,81 | 23,13 | 19,12 | 20,9 | 26,32 | 25,36 | 25,74 | 23,08 | 23,48 |  | 30,6 | 30,22 |
| BsPerR | 25,53 | 27,69 | 24,82 | 29,66 | 22,22 | 27,27 | 26,24 | 26,09 | 30,07 | 25,55 | 24,63 | 28,78 | 30,6 |  | 40,69 |
| SpPerR | 21,38 | 25,19 | 25,36 | 20,51 | 21,33 | 16,55 | 19,01 | 23,02 | 22,73 | 22,92 | 21,48 | 23,45 | 30,22 | 40,69 |  |

**Table S4:** Fur superfamily proteins aminoacidic sequence identity matrix. Analysis was performed with Clustal Omega (Chenna et al., 2003). More information in the excel file.
